# Adaptations to Endosymbiosis in a Cnidarian-Dinoflagellate Association: Differential Gene Expression and Specific Gene Duplications

**DOI:** 10.1371/journal.pgen.1002187

**Published:** 2011-07-21

**Authors:** Philippe Ganot, Aurélie Moya, Virginie Magnone, Denis Allemand, Paola Furla, Cécile Sabourault

**Affiliations:** 1 Université de Nice-Sophia-Antipolis, Nice, France; 2 Université Pierre et Marie Curie, Paris, France; 3 Centre National de la Recherche Scientifique, Roscoff, France; 4 UMR7138 Systématique, Adaptation, Evolution, Nice, France; 5 Institut de Pharmacologie Moléculaire et Cellulaire, UMR 6097, Sophia Antipolis, France; 6 Centre Scientifique de Monaco, Monaco, Monaco; University of California San Francisco, United States of America

## Abstract

Trophic endosymbiosis between anthozoans and photosynthetic dinoflagellates forms the key foundation of reef ecosystems. Dysfunction and collapse of symbiosis lead to bleaching (symbiont expulsion), which is responsible for the severe worldwide decline of coral reefs. Molecular signals are central to the stability of this partnership and are therefore closely related to coral health. To decipher inter-partner signaling, we developed genomic resources (cDNA library and microarrays) from the symbiotic sea anemone *Anemonia viridis*. Here we describe differential expression between symbiotic (also called zooxanthellate anemones) or aposymbiotic (also called bleached) *A. viridis* specimens, using microarray hybridizations and qPCR experiments. We mapped, for the first time, transcript abundance separately in the epidermal cell layer and the gastrodermal cells that host photosynthetic symbionts. Transcriptomic profiles showed large inter-individual variability, indicating that aposymbiosis could be induced by different pathways. We defined a restricted subset of 39 common genes that are characteristic of the symbiotic or aposymbiotic states. We demonstrated that transcription of many genes belonging to this set is specifically enhanced in the symbiotic cells (gastroderm). A model is proposed where the aposymbiotic and therefore heterotrophic state triggers vesicular trafficking, whereas the symbiotic and therefore autotrophic state favors metabolic exchanges between host and symbiont. Several genetic pathways were investigated in more detail: i) a key vitamin K–dependant process involved in the dinoflagellate-cnidarian recognition; ii) two cnidarian tissue-specific carbonic anhydrases involved in the carbon transfer from the environment to the intracellular symbionts; iii) host collagen synthesis, mostly supported by the symbiotic tissue. Further, we identified specific gene duplications and showed that the cnidarian-specific isoform was also up-regulated both in the symbiotic state and in the gastroderm. Our results thus offer new insight into the inter-partner signaling required for the physiological mechanisms of the symbiosis that is crucial for coral health.

## Author Summary

The global decline of reef-building corals is of particular concern: they are among the most threatened marine ecosystems. Coral reefs have been decimated over the last several decades, leading to a rapid loss of reef biodiversity and the loss of the resources that humans derive from them. Coral health depends mainly on the trophic intimate association between the animal host and the photosynthetic symbionts (dinoflagellates) residing within its cells. Insights into the physiological mechanisms that underlie healthy as well as stressed (or bleached) corals are thus critical to predict whether they will be able to adapt to and survive climate change. We used a transcriptomic approach to decipher the molecular dialogues between a cnidarian host and its dinoflagellate symbionts. We identified in the host new key pathways that contribute to the symbiotic interaction, as well as specific genetic adaptations.

## Introduction

The mutualistic symbiosis of anthozoans (Cnidaria), such as corals and sea anemones, with their intracellular dinoflagellate symbionts, mostly of the genus *Symbiodinium*, forms both trophic and structural foundation of coral reef ecosystems. Anthozoans have a very simple body plan and are composed of two tissue layers, the epidermis and the gastroderm (also called ectoderm and endoderm, respectively [1], Figure S1A). They host their unicellular symbionts, also called zooxanthellae, inside vacuoles (symbiosomes) within the gastrodermal cell layer. Safely localized inside the animal host cells, the photosynthetic symbionts fix large quantities of carbon dioxide. Most of the reduced organic carbon produced is then translocated to the host as mobile compounds, such as glycerol, lipids and amino acids [2]. In return, the host provides suitable conditions for symbiont photosynthesis: inorganic nitrogen, phosphorus and inorganic carbon, as well as a favorable high light environment [2].

This partnership generates many constraints, however, resulting in physiological and cellular adaptations (for review, [3], [4]). For example, the presence of photosynthetic zooxanthellae within the gastrodermal cells requires the host to transport inorganic carbon from the surrounding seawater to the symbionts, a process in which carbonic anhydrases (CAs) are central [5], [6]. To prevent possible cellular damage resulting from photosynthesis-induced hyperoxia, both partners also express a high diversity of antioxidant enzymes, including catalases, peroxidases and superoxide dismutases (SODs), [7]–[9]. SOD and CA isoforms specific to symbiotic anthozoans have been demonstrated [8], [10].

Environmental perturbations such as an increase in seawater temperature may induce dysfunction and collapse of the symbiosis, leading to zooxanthellae loss or so-called “bleaching”, and this phenomenon has led to severe worldwide decline of coral reefs [11]. The establishment and maintenance of this partnership must therefore be dependent on intimate molecular communications between the partners, including recognition and tolerance of symbionts, as well as adaptations for mutual transport and exchange of nutritional resources. A small number of candidate genes for this molecular dialogue has been proposed, including CAs and the cell adhesion protein Sym32 [10], [12], [13]. These have so far been examined by targeted protein analyses (for review, see [14]). Large-scale gene expression studies have tended to highlight a modulation of the host transcriptome, in particular genes involved in cell adhesion, lipid metabolism, cell cycle regulation, or cell death [15]–[17]. While most transcriptomic approaches have been performed in symbiotic cnidarians under thermal stress, imposed in order to understand the molecular and cellular basis of the early events leading to bleaching [18], [19], we rather focused our present experimental approach on two stable states: symbiotic and aposymbiotic individuals.

How do anthozoans maintain a stable partnership with their photosynthetic dinoflagellate symbionts? To decipher the molecular dialogue inferred by the presence of the dinoflagellate symbionts (*Symbiodinium* clade temperate A) within the sea anemone *Anemonia viridis*, we compared transcriptomes of symbiotic and aposymbiotic specimens using a symbiosis-dedicated microarray. This oligonucleotide microarray (2,000 features) was developed from the *A. viridis* 40,000 EST collection [20] and is dedicated to genes potentially involved in symbiosis regulatory pathways.

Our two main goals were to identify gene expression patterns characteristic of: i) the symbiotic and aposymbiotic conditions, ii) the epidermis and gastroderm tissue layers. The main advantage of our model, *A. viridis*, is it allows efficient separation of the two tissue layers of the animal (epidermis and gastroderm) with minimal cross contamination [8]. Concerted DNA microarrays and quantitative RT-PCR (qPCR) analyses outlined characteristic gene expression signatures for the symbiotic and aposymbiotic states. Among newly identified genes, some appeared to result from anthozoan-specific gene duplications. Analyses of tissue-specific expression demonstrated that most of the host genes involved in symbiotic interactions are preferentially expressed in the gastroderm (i.e. the zooxanthellate tissue layer). We detailed several genetic regulatory pathways involved in dinoflagellate-cnidarian recognition, carbon transfer to the intracellular symbionts and mesoglea constitution. Finally, we propose a model where the aposymbiotic state triggers vesicular trafficking whereas the symbiotic state favors metabolic exchanges between host and symbionts.

## Results

### Molecular quantification of dinoflagellates

The “bleached” phenotype is shared by many stressed symbiotic cnidarians and is the consequence of a massive upstream loss of symbionts and hence their photosynthetic pigments. Most approaches to quantify symbiont loss use manual zooxanthellae counts. Here, we developed a fast and accurate approach using real-time quantitative PCR on total genomic DNA extracts to quantify the relative number of nuclei and hence cells.

**Figure 1:**
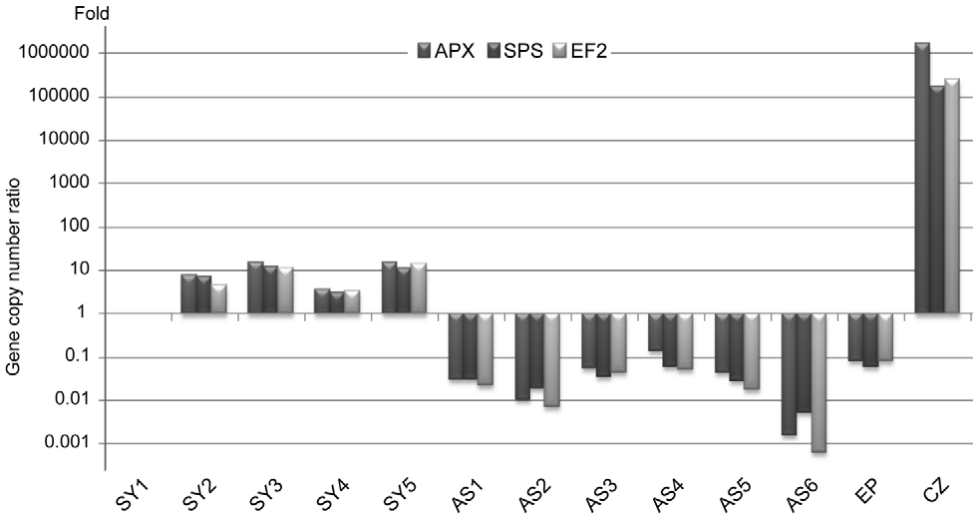
*Symbiodinium* quantification by real-time quantitative PCR (symbiont to host nuclei ratio). *Symbiodinium* nuclear EF2, SPS, APX and *A. viridis* nuclear COP-γ gene copy numbers were assessed by qPCR performed on total genomic DNA extracts from 5 symbiotic anemones (Sy1–Sy5), 6 aposymbiotic anemones (AS1–AS6), *in vitro* cultured *Symbiodinium* (CZ) and epidermal tissue fraction (Ep). The histogram represents the relative copy number ratios of EF2, SPS and APX to COP-γ, relative to the Sy1 sea anemone, expressed in fold number.

*Symbiodinium* (temperate A clade) nuclear Elongation Factor 2 (EF2), Sucrose Phosphate Synthase (SPS) and Ascorbate Peroxidase (APX) and the *A. viridis* nuclear Coatomer subunit gamma (COP-γ Regulator of Chromosome Condensation protein 2 (RCC2) and Niemann-Pick disease type C1 (NPC1) gene copy numbers were assessed for total genomic DNA extracts from 5 symbiotic anemones (Sy1–Sy5) and 6 aposymbiotic anemones (AS1–AS6) collected along the Mediterranean coasts around Nice, France (Figure S2) and kept in laboratory culture, as well as *in vitro* cultured *Symbiodinium* (CZ) and epidermal tissue fraction (Ep). Although gene locus number per nucleus is assumed to remain constant for a given species, dinoflagellates have been shown to undergo gene specific amplifications [21], [22]. In *Amphidinium carterae*, the EF2 gene is present in tandem repeat contrary to APX [21]. Whilst the 3 *A. viridis* gene loci were present in a nearly 1∶1 ratio in all specimens, in *Symbiodinium* the number of genes was relatively variable from one specimen to another despite the proximity of collection sites to one another. The SPS∶APX ratio was around 1∶1 in all specimens except for AS2 and AS6. EF2∶APX and EF2∶SPS ratios displayed strong polymorphism, likely due to EF2 variable gene amplification (Figure S3A). Gene copy numbers between *A.viridis* and its symbiont were also measured. Figure 1 shows the relative symbiont to host nucleus ratio for APX/COP-γ SPS/COP-γ and EF2/COP-γ (similar results were obtained with RCC2 and NPC1, Figure S3B). Whatever gene locus used, the pattern of symbiont to host cell ratio was reproducibly similar with ratios between aposymbiotic and symbiotic specimens ranging from 8.2×10^−2^ (AS4 *versus* Sy1) to over 10^−4^ fold (AS6 *versus* Sy5).

### Inter-individual variability in symbiotic and aposymbiotic expression profiles

In order to identify gene expression patterns characteristic of symbiosis, the gene expression profiles of symbiotic and aposymbiotic anemones were compared using a symbiosis-dedicated oligoarray. 60-mer oligonucleotides were designed from 2,000 sequences putatively involved in symbiosis selected from the large clustered and annotated *A. viridis* EST collection [20]. Figure S1 gives an overview of the Gene Ontology (GO) functional annotations of the selected sequences.

**Figure 2:**
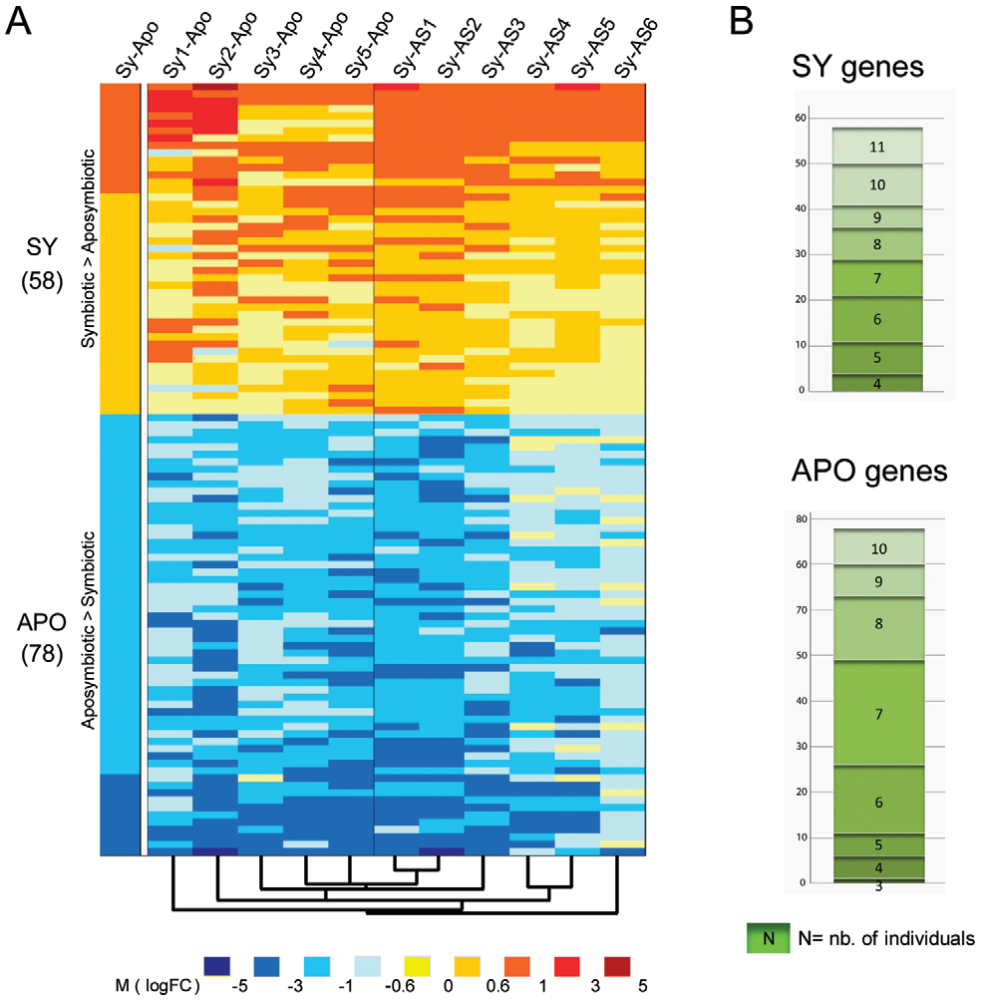
Differentially expressed genes (DEG) between symbiotic and aposymbiotic anemones. A. Heatmap diagram of DEG. Left column (Sy-Apo) represents expression of the 58 up- (SY) and 78 down-regulated (APO) genes (|M|>0.59, B>0) identified from the batch comparison of the 5 pooled symbiotic *versus* the 6 pooled aposymbiotic anemones. The following columns to the right represent expression of the same genes in each individual symbiotic anemone (Sy1–5) compared with the pooled aposymbiotic (-Apo) anemones and in each individual aposymbiotic anemone (AS1–6) compared with the pooled symbiotic (-Sy) anemones. The dendogram underneath the heatmap represents the cluster array tree result performed on the entire result dataset (with Cluster3). The color intensity code representing M value intervals is shown below. B. A given gene can be differentially expressed in the batch analysis (SY or APO) although only differentially expressed in few individual sea anemones. Histogram shows the number of individual anemones (3–11) each gene is differentially expressed in. Top and bottom panels: gene distribution of SY and APO genes, respectively.

Microarray experiments were performed between symbiotic and aposymbiotic anemones, using a dual-dye protocol, one condition being labeled with Cy3, and the second by Cy5. The experimental design of hybridizations is shown in Figure S4. Microarray results were analyzed according to two methods. First, in order to have an overall estimate of the genes differentially expressed between the two states, we treated the 11 individual specimens as two batch categories: symbiotic anemones (Sy1–Sy5) and aposymbiotic anemones (AS1–AS6). Statistical analysis was performed with the limmaGUI package [23] that defined for each gene in each experiment an average fold change (M, corresponding to a log2 ratio between the 2 experimental conditions) and a statistical value (called B, with positive values for the more significant variations). 58 and 78 genes were significantly up-regulated (|M|>0.59 and B>0) in the symbiotic (hereafter called “SY gene set”) and aposymbiotic states (hereafter called “APO gene set”), respectively (Figure 2A, Tables S1 and S2). Functional annotation of these 136 genes using GO terms and statistical analysis by Gossip (Fisher's exact test, p<0.05) showed that the terms “plastid”, “calcium ion binding”, and “protein modification process” were over-represented in the SY gene set, in contrast to “cytosol”, “cytoplasmic membrane-bounded vesicle” and “transcription”, which were over-represented in the APO gene set (Figure S1C).

Second, in order to gain insights about inter-individual variation in gene expression, we compared each individual symbiotic anemone (Sy1 to Sy5) with the batch of 6 aposymbiotic anemones (Apo), and inversely, compared each aposymbiotic anemone (AS1 to AS6) with the batch of 5 symbiotic anemones (Sy). This analysis (Figure 2A) showed unexpectedly high inter-individual variability of differential gene expression patterns within these 136 genes. Figure 2B shows the number of individual anemones for which a given gene is significantly differentially expressed between the SY and APO gene sets. For instance, only 8 SY genes were differentially expressed in all 11 anemones. Conversely, one APO gene (M = −0.63; B = 1.15) encoding for Interferon Regulatory Factor 1 was significantly differentially expressed in only 3 out of 11 anemones. To obtain an indication of the source of variability, we clustered the different anemones according to their expression. Different clustering parameters were tested and showed similar cluster trees; the most representative is shown underneath the heatmap (Figure 2A). Whatever the parameters used, specimens Sy4 and Sy5, AS1 and AS2, and AS4 and AS5 always paired together. Remarkably, even if the anemones were maintained in the same culture conditions at least 3 months before sampling, such co-clustering seemed to correlate with their previous life history: different collection dates/areas for symbiotic specimens, and different bleaching causes for aposymbiotic sea anemones (see Material and Methods and Figure S2). Nonetheless, these variable transcriptomic profiles define the same stable symbiotic or aposymbiotic phenotype.

### Definition of the Kern gene subset

The “Kern” gene subset (named after the German word for nucleus) was where the remainder of the analysis was focused, and is defined as those genes of cnidarians origin only (excludes genes of unicellular or prokaryotic origin) found in the SY or APO gene set, and differentially expressed in at least 8 out of the 11 specimens. Table 1 lists the representative 19 and 20 Kern genes found in the SY and APO gene sets respectively. A blast homology search against the *Nematostella vectensis* genome database and the Uniprot generalist database, and searches for specific protein signatures (signal peptide, trans-membrane domains or others domains) allowed the genes to be named and functions and cellular localizations assigned (Table 1). Among the different functional categories found, cell adhesion proteins (8/39) were the most represented, a process indeed expected to play a key role in signaling events between partners. A total of 7 other genes were involved in metabolism (4 SY and 3 APO). The four SY genes were specifically involved in fatty acid metabolism, indicating that fatty acid metabolism is likely a preponderant metabolic pathway of the symbiotic condition. Although all genes listed in Table 1 would merit further investigation, only several selectively targeted genes are detailed in this study (see below).

**Table 1:** Kern set of symbiotic genes.

	Av_Cluster	Sign. Pep.	TM	Expected cell localization*	Note	Protein Name	Expected role	Array Fold	qPCR Fold
1	av01015l05	Nv		ER	EF hand, Ca^2+^ binding and Vitamin K cycle	Calumenin precursor (Calu-a)	Ca^2+^ binding	7.29	
2	CL1319Ct1	SP		End, Lys	Cholesterol transport	Niemann Pick type C2 protein homolog (NPC2-D)	metabolism-FA	4.39	4.6
3	CL4283Ct1			Cyt	CO_2_ conversion; pH regulation	Carbonic anhydrase 2 cytosolic (CA2-c)	pH homeostasis	4.06	13.2
4	CL363Ct1	SP	TM	Out. pl. mb	Cell adhesion/recognition_fasiclin ×2	Sym32	cell adhesion	3.36	47.8
5	CL263Ct1	SP	TM	Out. pl. mb	CO_2_ conversion; pH regulation	Carbonic anhydrase 2 membrane (CA2-m)	pH homeostasis	2.90	8.6
6	av01009g10	N.v.		Sec	Cell adhesion/recognition, bacterial defense	Uromodulin domain (URO domain)	cell adhesion	2.66	
7	av02058f16	SP		ECM	No collagen domain detected in sequence	Col protein	cell adhesion	2.53	
8	av01002j09	N.v.	N.v.	Trans-mb.	Ig-like domain; Tyrosine-protein kinase	Tyrosine kinase receptor (Tyr-K Receptor)	receptor signaling	2.35	
9	av02120i19			Inn. pl. mb	Intracellular signalling; cytoskeletal regulation	Catenin delta	cell adhesion	2.17	
10	CL1994Ct1		TM	Mit, Per	Mitochondrial fission	Mitochondrial fission 1 protein (FIS1)	vesicle	1.98	
11	av02092k17			Cyt	Anti-apoptotic	E3 ubiquitin-protein ligase RNF34	Ubiquitin pathway	1.87	
12	CL3310Ct1	N.v.		Sec	Oxidative defense	Secretory glutathione peroxidase	antioxidant	1.85	
13	CL298Ct1			lipid droplet	Lipid binding; Regulation. of FA & steroid metabolism	Lipid storage droplets surface-binding protein 2	metabolism-FA	1.82	
14	av02096o03	N.v.	N.v.	Out. pl. mb	Collagen production	Prolyl-4-hydroxylase-alpha	Collagen processing	1.82	
15	av01005l13	N.v.	N.v.	Per	Fatty acid alpha oxidation (Plant spec.)	2-hydroxyacyl-CoA lyase 1	metabolism-FA	1.81	
16	av01017f01			Cyt	EF hand, Calcium binding	Visinin-like protein 1	Ca^2+^ binding	1.79	
17	av02097k23			Cyt	Terpenoid metabolism (SDR)	Dehydrogenase/reductase_SDR family member 12	metabolism-FA	1.72	
18	av01023c17			Cyt	Ubiquitin carboxyl-terminal hydrolase 34	Ubiquitin thioesterase 34 (Ubiquitin hydrolase 34)	Ubiquitin pathway	1.71	
19	CL187Ct1	SP	TM	Out. pl. mb	ZP domain recognised in receptor-like glycoproteins	Zona Pellucida-like domain-containing protein	cell adhesion	1.58	
20	CL1460Ct1			Cyt	Catabolism (first step) of the essential AA (L, I, V)	Branched-chain-amino-acid aminotransferase	metabolism	−1.58	
21	CL2889Ct1			Cyt	Dopamine metabolic process	E3 ubiquitin-protein ligase (parkin)	Ubiquitin pathway	−1.59	
22	av01029c01			Cyt	TRAPPC2 vesicle-mediated transport (ER to golgi)	Trafficking protein particle complex I sub.2 (Sedlin)	vesicle	−1.60	
23	CL2824Ct1			Cyt	TRAPPC10 vesicle-mediated transport (trans-Golgi)	Trafficking protein particle complex II sub.10 (TMEM1)	vesicle	−1.63	
24	CL829Ct1	SP		Sec	Oxidative defense, extracellular matrix, phagocytosis	Peroxidasin	antioxidant	−1.68	
25	CL1127Ct1			Cyt	Ion transport & cell volume	STE20	ion transport	−1.69	
26	CL2771Ct1	SP	TM	Phag	Ion transport & pH regulation, phagocytosis	Voltage-gated Hydrogen channel 1 (HVCN1)	ion chanel	−1.74	
27	av02120i05	Gal.		Sec	Extracellular matrix and cell surface receptor proteins	Galaxin	cell adhesion	−1.76	
28	CL240Ct1			Cyt	Gluconeogenesis (rate-controlling 1st step via pyruvate)	Phosphoenolpyruvate carboxykinase (PEPCK-C)	metabolism	−1.81	
29	av02125c09	N.v.	N.v.	End	Metabolism of iodometabolites; inactivation of T4 and T3	Type III iodothyronine deiodinase	metabolism	−1.82	
30	av02109e14	N.v.	Hyd.	Sec	Oxidoreductase; source of ammonium	Polyamine oxidase	antioxidant	−1.83	
31	av02115o13			Nu, Cyt	Serine/threonine-protein kinase, PI3/PI4-kinase family	Ataxia telangiectasia and Rad3 related (ATR)	DNA damage	−1.89	
32	CL2461Ct1			Cyt	Ubiquitin ligase	Cullin family	Ubiquitin pathway	−1.90	
33	av01046b22			Nu	pre-mRNA splicing	pre-mRNA-splicing factor 18; (PRP18)	RNA processing	−2.02	
34	av02084n10			Nu, Cyt	Rev.transcriptase; Retrotransposon	Putative transposon-derived protein ReO_6	transposon	−2.12	
35	av02100k07			Cyt	Detoxification reactive oxygen speciess	Glutathione S-transferase Y1; (GST class-mu)	detoxification	−2.15	
36	av02069c14	N.v.	N.v.	End, Lys	Homeostasis; ion transport	SLC30a2, Zinc transporter 2 (ZnT-2)	solute transporter	−2.24	
37	av01039o13	N.v.		Sec	Can interact with lectins	C3-2 complement	cell adhesion	−2.25	
38	CL627Ct1			Inn. pl. mb	Clathrin coat assembly protein	AP-2 sigma-1	vesicle	−2.40	
39	av02114d05	Hyd.		Sec	EGF-like domains; adhesion	Notch-like family	cell adhesion	−4.60	

Genes differentially expressed (|M|>0.59, B>0) in at least 8 individuals out of our 11 tested anemones were defined as belonging to the “Kern” set of symbiotic genes. Each corresponding Uniseq (Av_Cluster) was subjected to a blastX homolog search and analyzed for signal peptide, trans-membrane domain and other protein signatures. When the *A. viridis* sequence was partial, the *N. vectensis* (Nv) or *Galaxea fascicularis* (gene # 27) ortholog was taken into account. From homolog function and protein signature analysis, putative cellular localization and function were assigned. Fold gene expression (symbiotic *versus* aposymbiotic sea anemones) was determined experimentally from microarrays and qPCR experiments.

*expected sub-cellular localisation: Cyt, cytoplasm; ECM., extracellular matrix; ER, Endoplasmic Reticulum; End, Endosome; Lys, Lysosome; Mit, Mitochodrion; Nu, Nucleus; Per, Peroxisome; Phag, Phagosome; pl.mb. (out. or inn.), plasma membrane (outer or inner); Sec, Secreted; Trans-mb, Trans-membrane.

**Figure 3:**
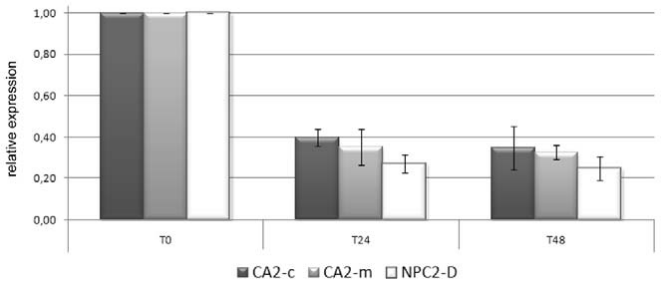
CA2-m, CA2-c, and NPC2-D expression in response to imposed thermal stress. Expression of CA2-m, CA2-c and NPC2-D after 0, 24 h and 48 h of 8°C heat stress was measured using RT-qPCR. Three anemones (Sy3–5) were assayed. Gene expression, normalized to RCC2 and COP-γ, is given related to t0. Error bars represent standard error.

In order to obtain support for the functional implication of some of the Kern genes, we monitored the expression of genes of interest directly after thermal stress, which is known to disrupt symbiosis. Anemones Sy3–5 were subjected to an 8°C temperature increase, and the expression of CA2-c, CA2-m and NPC2-D dropped by around 3 fold after 24 h and 48 h (Figure 3). This result is interesting, considering their up-regulation in symbiotic individuals. This immediate response to environmental stress precedes symbiosis breakdown and thus strengthens support for the possible role of these 3 genes in endosymbiosis in *A. viridis*.

### Gastroderm-specific gene expression

**Figure 4:**
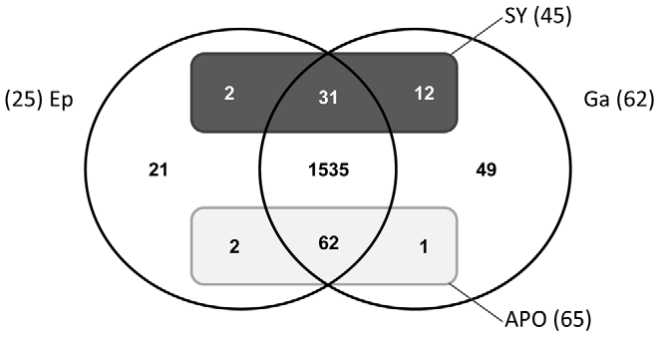
Venn diagram of genes expressed in sea anemone tissues. Distribution of genes (only of cnidarian origin) expressed in the epidermis (Ep) or gastroderm (Ga) compartments (circles), relative to their up-regulation (rectangles) in symbiotic (SY) or aposymbiotic (APO) conditions.

**Figure 5:**
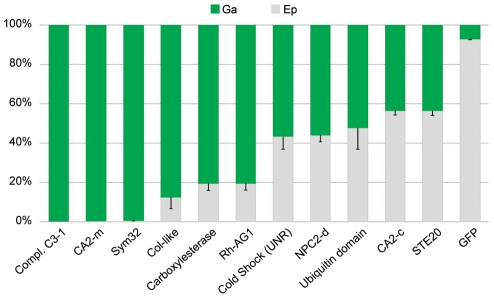
Tissue-specific expression of targeted genes. RT-qPCRs were performed on the epidermal (Ep) and gastrodermal (Ga) total RNA extracts from 3 different sea anemones. The histogram shows the relative Ep (gray) *versus* Ga (green) expression of the genes listed on the histogram (error bars represent standard error).

As the two tissue layers can be separated in *A. viridis*, it is a powerful biological model for studying tissue-specific gene expression. We used two experimental designs to compare gastrodermal *versus* epidermal gene expression profiles (Figure S4). Firstly we directly hybridized the cDNAs of the two tissues against each other on the same array, and secondly we compared each tissue sample to the aposymbiotic AS6 reference and thus defined tissue expression profiles by transitivity. Only the genes with at least 1.50 fold increase in transcript abundance (M>0.59) in one tissue in both experiments were assigned as differentially expressed (Table 2, Tables S1 and S2). As expected, the expression of most *Symbiodinium* genes was restricted to the gastroderm. The Venn diagram in Figure 4 summarizes distribution of the 1,715 cnidarian-specific genes (prokaryote and zooxanthellae genes were excluded from the analysis) according to their gastrodermal (Ga), epidermal (Ep), and preferential symbiotic (SY) *versus* aposymbiotic (APO) expression. We further confirmed preferential expression of several genes in the different tissues using real-time quantitative PCR (Figure 5). Taken together, microarray and qPCR results showed that many more *A. viridis* genes were preferentially expressed in the gastroderm (71%) than in the epidermis (29%). More interestingly, among the 17 genes which were both preferentially expressed in a given tissue and differentially regulated under symbiotic/aposymbiotic status, the large majority (12) were in fact up-regulated within the gastroderm of symbiotic anemones (*i.e.* the zooxanthellate tissue). This suggests that the presence of symbionts directly modulates the gene expression of their hosting gastrodermal cells. In addition, out of these 12 genes, 9 belong to the Kern subset of genes, supporting the importance of this gene set in inter-partner communication and regulation (Figure 5 and Table 2). Hence, we have identified different categories of genes potentially involved in sequential aspects of symbiosis, and showed for the first time that the animal tissue-specific molecular response to the presence/absence of zooxanthellae was restricted almost entirely to preferential expression of genes within the compartment hosting the symbionts. These results support the crucial role of the gastroderm in this symbiotic interaction.

**Table 2:** Examples of *A. viridis* genes up-regulated in the epidermis (E) or gastroderm (G).

Av Cluster	Expect. cell localization	Protein Name	Expected Role	DEG	Fold Sym/Apo		Localization	
					Array	qPCR	Array	qPCR
av01015l05	ER	Calumenin precursor (AvCALUa)	Ca^2+^ binding	1 #	7.29		**G**	
CL1014Ct1	ER	Calumenin precursor (AvCALUb)	Ca^2+^ binding		1.15			
CL101Ct1	ER	Calumenin precursor (AvCALUc)	Ca^2+^ binding		−1.27			
CL363Ct1	Out. pl. mb	Sym32	cell adhesion	4 #	3.36	47.9	**G**	**G**
av02077g18	Out. pl. mb	Periostin	cell adhesion		1.36			
CL1319Ct1	End	Niemann Pick type C2 protein homolog NPC2-D	metabolism-FA	2 #	4.39	4.6	**G**	**G**
CL214Ct1	End	Niemann Pick type C2 protein homolog NPC2-a	metabolism-FA		−1.06			
CL308Ct1	End	Niemann Pick type C1 protein homolog NPC1	metabolism-FA		1.13			
CL263Ct1	Out. pl. mb	Carbonic anhydrase 2 membrane (CA2-m)	pH regulation	5 #	2.90	8.6	**G**	**G**
CL4283Ct1	Cyt	Carbonic anhydrase 2 cytosolic (CA2-c)	pH regulation	3	4.06	13.2		**ge**
av01043f14	Pl. mb.	Rh AG 1	solute transporter	#	1.58	4.4		**G**
av02095l23	Pl. mb.	Rh AG 2	solute transporter		1.11			
CL506Ct1	Sec	C3-1 complement	cell adhesion		1.47	1.3		**G**
av01039o13	Sec	C3-2 complement	cell adhesion	35	−2.25			
CL92Ct2	Sec	MERP-1	cell adhesion		−1.57			
CL194Ct2	Sec	MERP-1	cell adhesion		−1.37		**G**	
CL51Ct1	Sec	MERP-1	cell adhesion		−1.06		**G**	
av02096o03	Pl. mb.	Prolyl-4-hydroxylase alpha	collagen processing	14 #	1.82		**G**	
CL3699Ct1	Pl. mb.	Prolyl-4-hydroxylase alpha	collagen processing		−1.00		**G**	
av02049b06	ER	Procollagen lysine2 oxoglutarate 5 dioxygenase 3	collagen processing		1.24			
av02062m24	ECM	Collagenase type IV	collagen processing		−1.08			
CL143Ct1	ECM	Collagenase type IV	collagen processing		−1.39			
CL38Ct2	ECM	Collagen-like	collagen precursor		−1.07		**G**	
CL1481Ct1	ECM	Collagen-like	collagen precursor		−1.09		**G**	
av01033k13	ECM	Collagen, type IX, alpha 1	collagen precursor		−1.10			
av02101l17	ECM	Collagen like	collagen precursor		−1.26		**G**	
CL2177Ct1	ECM	Collagen alpha-5(VI)	collagen precursor		1.20		**G**	
av01030e14	ECM	Collagen alpha-2(V)	collagen precursor		−1.34		**G**	
CL253Ct1	ECM	Collagen alpha-2(I)	collagen precursor		1.15		**G**	
CL902Ct1	ECM	Collagen alpha-2(I)	collagen precursor		−1.06		**G**	
CL2629Ct1	ECM	Collagen alpha-1(V)	collagen precursor		−1.03		**G**	
CL635Ct1	ECM	Collagen alpha-1(V)	collagen precursor		−1.23		**G**	
CL389Ct1	ECM	Collagen alpha-1(III)	collagen precursor		−1.19		**G**	
CL4Ct6	ECM	Collagen alpha-1(II)	collagen precursor		−1.36		**G**	
CL934Ct1	ECM	Col protein	collagen precursor		−1.02		**G**	
av02058f16	ECM	Col-like protein	cell adhesion	7 #	2.53			**G**
av01028j14	ECM	Collagen triple helix repeat-containing protein 1	cell adhesion		−1.23		**E**	
av01009g10	Sec	Uromodulin domain (URO domain)	cell adhesion	6 #	2.66		**G**	
av02120i19	Inn. pl. mb	Catenin delta	cell adhesion	9 #	2.17		**G**	
CL1994Ct1	Mit, Per	Mitochondrial fission 1 protein (FIS1)	vesicle	10 #	1.98		**G**	
av02071b13	Nu	Tousled-like kinase 1	cell cycle	#	1.82		**G**	
CL3005Ct1	Nu	DNA repair protein RAD50	DNA damage	#	1.60		**G**	
av02071o11	Cyt	Cytoskeleton-associated protein 5 (TOG protein)	cytoskeleton	#	−1.52		**G**	
av01002j09	Pl. mb.	Tyrosine kinase receptor (Tyr,K Receptor)	receptor signaling	8 #	2.35		**E**	
av02074d19		Sulfuric ester hydrolases	hormone	#	1.63		**E**	
CL2622Ct1		Predicted protein	receptor signaling	#	−1.59		**E**	
CL3901Ct1		Hydroxymethylglutaryl-CoA reductase	metabolism-FA	#	−1.73		**E**	
av01020d21		Carboxylesterase	metabolism-FA		−1.53	2.0		**ge**
CL450Ct1	Nu-Cyt	Cold shock domain-containing protein E1 (UNR)	RNA processing		−1.70	−1.3		**ge**
CL1958Ct1		no hits found	predicted prot		−1.03	−1.9		**ge**
CL1127Ct1	Cyt	STE20	ion transport	23	−1.69	−1.4		**ge**
CL612Ct1	Cyt	GFP	GFP		1.23	1.5	**E**	**E**

Genes are grouped by family or common function. Fold (symbiotic minus aposymbiotic anemones) and Tissue-specific (E: Epidermis; G: Gastroderm+Zoox; ge: E+G) gene expressions were determined experimentally from microarray and qPCR experiments. Legend is as in Table 1.

Expected sub-cellular localization: Cyt, cytoplasm; ECM, extracellular matrix; ER, Endoplasmic Reticulum; End, Endosome; Lys, Lysosome; Mit, Mitochodrion; Nu, Nucleus; Per, Peroxisome; pl.mb., plasma membrane (outer or inner); Sec, Secreted; Trans-mb, Trans-membrane.

DEG: # gene is both differentially expressed between aposymbiotic *versus* symbiotic state and gastroderm *versus* epidermis; numbers refer to the Kern gene nomenclature as in Table 1.

The gastroderm-specific gene expression disclosed interesting hallmarks. Carbonic anhydrases are primordial enzymes necessary for the transport of inorganic carbon through biological membranes. Two isoforms were identified in our library and had increased transcript abundance in zooxanthellate anemones. Based on their respective protein signatures and blast homology, one isoform (Av_CA2-c, Kern # 3) is cytoplasmic whereas the other isoform (Av_CA2-m, Kern # 5) probably localizes to the outer plasma membrane, since it contains a signal peptide sequence as well as a trans-membrane and GPI anchor domains at its NH_2_ and COOH termini, respectively. The cytoplasmic CA2 was equally distributed in the two compartments, but the membrane-anchored CA2 was principally expressed in the gastroderm (Figure 5). Another interesting finding on the tissue distribution of gene expression was that collagen biosynthesis was broadly under the control of the gastroderm. In cnidarians, fibrillar collagen genes were recently shown to be much more represented than first expected from bilaterian evolutionary comparisons, with 8 different genes present in the genome of *N. vectensis* [24]. In *A. viridis*, we found 13 different cDNAs corresponding to portions of the *N. vectensis* homologs. Additionally, we monitored the expression of 5 genes regulating the post-translational processing of collagen synthesis. Interestingly, 14 of these cDNAs showed increased transcript abundance in the gastroderm (Table 2). The mesoglea, the acellular layer between epidermis and gastroderm, which mostly consists of collagen, may partly originate from the gastroderm in *A. viridis*. However, none of these genes showed differential expression between aposymbiotic and symbiotic states, except for Prolyl-4-hydroxylase alpha. This enzyme is a key chaperone for the biosynthesis of collagen, catalyzing the hydroxylation of proline residues of procollagen chains necessary for their correct three dimensional folding [25]. Two different isoforms were identified in our sequence library and the expression of one of them (Kern # 14) was specifically enhanced in zooxanthellate anemones. Thus, in addition to showing that the gastroderm synthesized much of collagen fibers, our results infer that *Symbiodinium* may exert, directly or indirectly, post-translational control on collagen synthesis and modulate the formation of the mesoglea in *A. viridis*.

### Cnidarian-specific gene duplication of symbiosis-related genes

**Figure 6:**
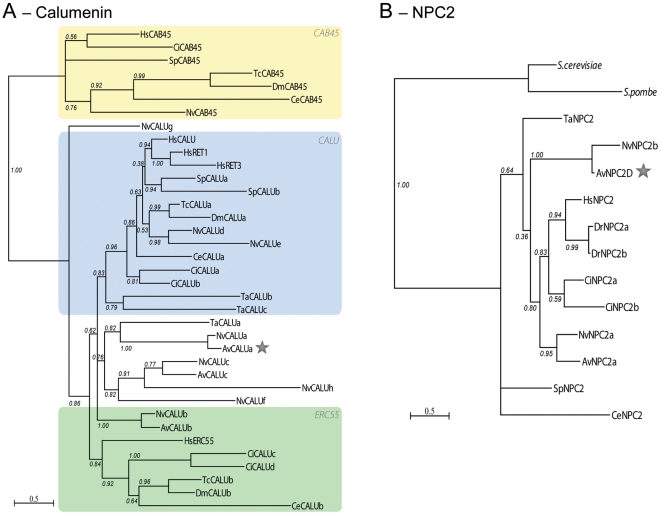
Gene duplication of NPC2 and Calumenin in anthozoans. Calumenin (A) and NPC2 (B) homologs alignments (Figure S5 and S6) were subjected to PhyML maximum likelihood phylogenetic analyses. Best-fitted substitution models were: [LG+I+G+F; I = 0.029 F = empirical and gamma = 1.127] for Calumenin and [WAG+I+G; I = 0.036 and gamma = 5.836] for NPC2. Hs: Human, Dr: *Danio rerio*, Ci: *Ciona intestinalis*, Sp, *Strongylocentrotus purpuratus*, Tc: *Tribolium castaneum*, Dm: *Drosophila melanogaster*, Ce: *Caenorhabditis elegans*, Nv: *Nematostella vectensis*, Av: *Anemonia viridis*, Ta: *Trichoplax adherans*, S.cerevisiae and S.pombe: NPC2 homologs in *Saccharomyces cerevisiae* (Q12408) and *Schizosaccharomyces pombe* (Q9C0X9), respectively. Protein sequences are given in Table S4. The star points to the *A.viridis* isoform belonging to the Kern that is up-regulated in the gastrodem of symbiotic anemones.

**Figure 7:**
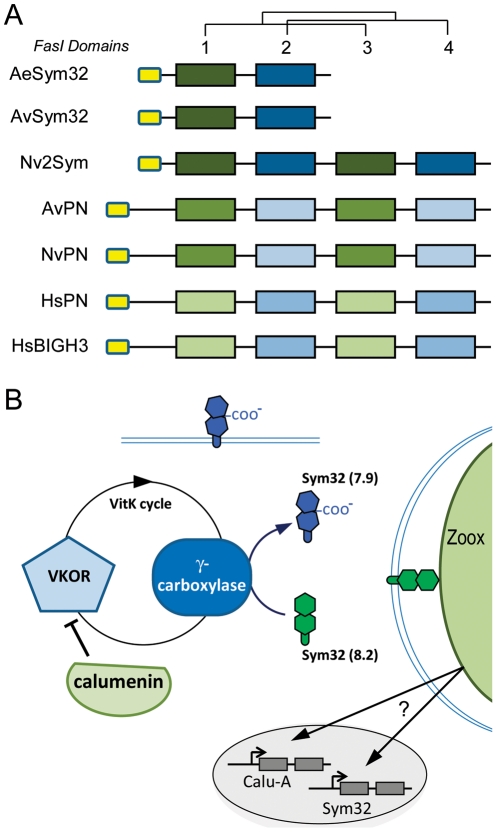
Sym32 gene duplication and putative γ-carboxylation regulation model. A. Color code schematization of the PhyML tree for the FasI domains of the Sym32, Periostin (PN) and BGH3 homologs in sea anemones (Ae, *A. elegantissima*; Av, *A. viridis*; Nv, *N. vectensis*) and human (Hs) (see tree in Figure S7b). Each rectangle represents one FasI domain (small yellow rectangles represent the Signal Sequence). FasI domains 1 and 3 are closely related. The first FasI domains of [AeSym32-1, AvSym32-1 and Nv2Sym-1 and -3] are closely related to each other and to a lesser extent with [AvPN-1 and -3, and NvPN-1 and -3] and finally with [HsPN-1 and -3, and HsBGH3-1 and -3]. A similar relationship exists for the FasI domains 2 and 4. AeSym32, AvSym32 and Nv2Sym are probable orthologs, except that Sym32 appears as the first half of Nv2Sym. The putative ortholog of HsPN is NvPN. B. Heuristic Model. The presence of symbionts activates the expression of calumenin and sym32 genes via an unknown mechanism. The CRS of the Sym32 protein is recognized as substrate by the activated vitamin K cycle (vitamin K is a cofactor produced from the photosynthetic organism) and in turn is γ-carboxylated. Meanwhile, the Calumenin represses the VKOR protein, inhibiting the γ-carboxylase. Two forms of Sym32 are thus expected to be produced from this pathway: the Glu-Sym32 and Gla-Sym32 electrophoretypes (likely corresponding to the two spots PI = 8.2 and PI = 7.9, respectively [51]). Only Sym32 (PI = 8.2) is found to be associated with the symbiosome membrane, underlying a novel functionality for Sym32 and γ-carboxylation.

During the course of our analysis, we noticed that some of the genes involved in symbiosis (Kern genes) had multiple paralogs in our dataset. Such was the case with the previously described MERP gene family [20], but the same held true for other Kern genes including Niemann Pick type C2 (NPC2), Calumenin, Sym32 and C3 Complement (C3) families (Table 2). We conducted phylogenetic analyses on the NPC2, Calumenin and Sym32 gene families in order to assign an evolutionary origin for each member. *A. viridis* isoforms present in our dataset were compared to their homologs in *N.vectensis* and other representative eukaryotes with a complete genome sequence. Both maximum likelihood (Figure 6 and Figure 7A) and Bayesian (Figures S5, S6, S7) methods gave very similar trees.

In vertebrates, Calumenin belongs to the CREC gene family, which encompasses 5 members (CAB45, ERC-55, Reticulocalbin 1 and 3, and Calumenin) [26]. From phylogenetic analysis across metazoans (Figure 6A), 3 groups of homologs can be defined; the CAB45 homologs, the ERC55 homologs and the CALU (Calumenin, Reticulocalbin 1 and 3) homologs. Cnidarian homologs are found within these 3 groups, however additional cnidarian homologs (NvCALUa,c,f–h, AvCALUa,c) are also present outside the CALU and ERC55 groups, representing cnidarian specific CALU gene duplications.

NPC2 (Figure 6B) is a single copy gene in human, nematode, sea urchin, Placozoa and yeast and is independently duplicated in ascidian and fish. Two copies of the NPC2 gene were found in sea anemones. Phylogenetic analysis supports a gene duplication in Anthozoans, with NvNPC2a and AvNPC2a representing the orthologs of the chordate NPC2, and NvNPC2b and AvNPC2-D defining an anthozoan specific duplication.

In the symbiotic sea anemones *A. viridis* and *Anthopleura elegantissima*, Sym32 is composed of two adjacent FasI domains (Figure 7A). In *A. viridis*, we identified two FasI-containing proteins: Sym 32 and the related periostin gene (PN), which is conserved across metazoans (only cnidarians and human are shown). AvSym32 and AvPN are composed of 2 and 4 FasI domains, respectively. In *N. Nematostella*, there are also two comparable FasI-containing proteins; both are composed of 4 FasI domains. One corresponds to the PN homolog (NvPN) and the second, which we named Nv2Sym, is similar to a tandem duplication of the AvSym32 sequence with 2×2 FasI domains. In human, two related FasI-containing proteins are characterized: the cognate Periostin (HsPN) and the “Transforming growth factor-beta-induced protein ig-h3” precursor (HsBGH3), both with 4 FasI domains. As proteins did not have the same number of FasI domains, we conducted a phylogenetic analysis on the alignment of all single FasI domains in order to gain insight into the domain evolution of this protein family (Figure 7A and Figure S7B). Human and cnidarian PN genes likely evolved from a common ancestor while Hs_PN and Hs_BGH3 would have duplicated after the Cnidaria - Bilateria separation. Consequently, AvSym32 and Nv2Sym are cnidarian-specific genes. Whether a Sym32 version containing only 2 FasI domains is specific to symbiotic anthozoans remains to be clarified.

Comparison of the expression of the *A. viridis* CALU, NPC2 and sym32 homologs showed that the isoform which was more highly expressed in the symbiotic state was also preferentially expressed in the gastroderm, whereas the other members were ubiquitously expressed (Table 2). For instance, AvCalu-a (Kern # 1) was the most up regulated gene in the symbiotic condition and localized in the gastroderm, whereas expression of both the other isoforms (AvCalu-b and AvCalu-c) was neither different between symbiotic *versus* aposymbiotic conditions nor between the tissue layers (Table 2). The same held true for the two isoforms of NPC2 (AvNPC2-D (Kern # 2) *versus* AvNPC2-a) and for Sym32 (Kern # 4) *versus* the Periostin homologs (Table 2). Most interestingly, the isoform that was differentially expressed was always member of the Kern gene set.

Thus, analysis of *N. vectensis* gene families identified cnidarian-specific gene duplications (NvCALUa,c,f–h, NvNPC2b, Nv2sym) and *A. viridis* ortholog analysis showed that among the cnidarian-specific isoform, the Kern genes (AvCALUa, AvNPC2-D and AvSym32) were up-regulated both in symbiotic state and in the gastroderm.

## Discussion

**Figure 8:**
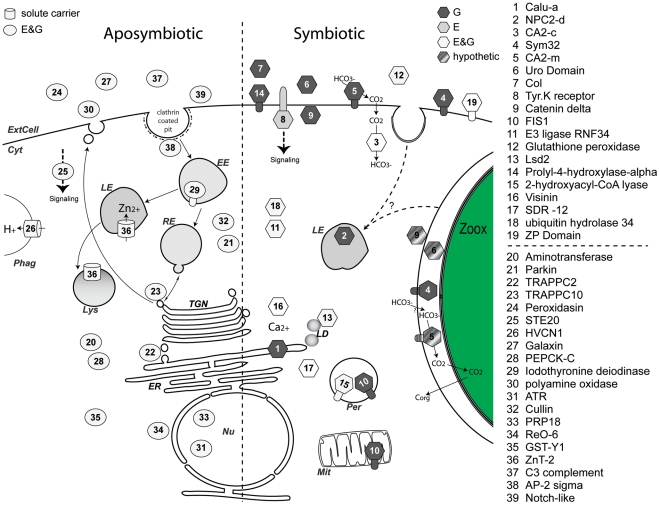
Model of pathways highlighted in the aposymbiotic and symbiotic states of sea anemones. Based on functional homology and protein signature (Table 1), each gene product from the Kern set was assigned a specific sub-cellular compartment or secretion pathway: ExtCell; Extra Cellular milieu; Cyt, Cytosol; Nu, Nucleus; TGN, Trans Golgi Network; ER, Endoplasmic reticulum; LD, Lipid droplets; Mit, Mitochondrion; Per, Peroxisome; Lys, Lysosome; Phag, Phagosome; EE, Early Endosome; RE, Recycling Endosome; LE, Late Endosome; Zoox, Zooxanthellae. The left and right moieties of the diagram show genes with increase transcript abundance in aposymbiotic and symbiotic states, respectively. Circles (aposymbiotic), barrels (solute carrier), and hexagons (symbiotic) represent gene expression specific to epidermis (E, dark grey), gastroderm (G, light grey) or both (E&G, white). Trans-membrane domains are shown by appendages. Each gene number has the corresponding name indicated in the list on the right. Gene products in dashed grey are candidate proteins that could potentially (hypothetic) be present at the perisymbiotic space.

Many anthozoans rely on photosynthetic endosymbionts (mostly *Symbiodinium* sp.) to grow in oligotrophic environments. The symbiotic relationship involves regulatory crosstalk between partners that allows the association to persist. This interpartner communication includes: i) the recognition of the partners, ii) the ability of symbionts to colonize host cells without being rejected by the host immune system, iii) the regulation of symbiont population, and iv) adaptations for mutual transport and exchange of nutritional resources [27]. In order to identify the genes potentially involved in the molecular dialog supporting this endosymbiosis, we used a microarray approach to compare the gene expression profiles of 11 *A. viridis* anemones representative of the symbiotic and the aposymbiotic states. We identified a subset of *A. viridis* genes, which we named “Kern”, characteristic of the symbiotic (SY) and aposymbiotic (APO) states in the sense that they were preferentially expressed in symbiotic or aposymbiotic anemones. Consequently, the Kern genes are potentially important candidate genes for the maintenance of the symbiosis process. Since in *A. viridis*, the two tissue layers can be separated, we were also able to assign tissue-specific gene expression. Many genes or gene functions that were expressed more highly in symbiotic anemones were also expressed more highly in the gastroderm, the symbiont hosting tissue. Functional annotation associated with sub-cellular localization of the Kern gene products allowed us to draw a map of the 39 genes differentially regulated in the symbiotic and aposymbiotic states (Figure 8).

### Autotrophy and heterotrophy

One of the first conclusions from our present model is that many genes that are present in higher abundance in the aposymbiotic state are associated with active vesicle trafficking, both in the endocytotic and secretory pathways, as opposed to the many membrane-bound proteins associated with cellular recognition and adhesion coded by genes in higher abundance in the symbiotic state (Table 1 and Figure 8). Under aposymbiotic conditions, transcription of several proteins controlling trafficking was markedly enhanced. These include TRAPPC2 and TRAPPC10 (Kern # 22 and 23), which are members of the TRAPP complexes that regulate endoplasmic reticulum (ER) to post-golgi vesicular trafficking [28], thus attesting for more active *de novo* biosynthesis in the aposymbiotic anemone than in the symbiotic host. As part of the same line of evidence, the highly differentially expressed gene AP-2 sigma-1 (Kern # 38) codes for a key protein in the formation of clathrin-coated vesicle [29], implying increased endocytic trafficking. Among other aposymbiotic marker genes, HVCN1 and SLC30a (Kern # 26 & 36) are two ion channels shown to be present in phagosomes and late endosomal/lysosomal vesicles, respectively [30], [31]. Of note, our dedicated array comprises several oligonucleotide probes for various rab mRNAs (including rab1a, 2a, 7a, 8a and 11a). Rab proteins have been shown to control vesicular trafficking in various species including symbiotic anemones [32]–[34] where the symbionts have been shown to detour host membrane trafficking resulting in failure of host lysosomes fuse with the symbiosomal membrane [34]. However, in our array results, none of the Av_rab genes were differentially expressed. This does not preclude a role of these Rab proteins in *A. viridis* symbiosis, as expression regulation may be at the translational level. Interestingly, the fact that vesicular trafficking appears to be much more active in aposymbiotic *A. viridis* corroborates with heterotrophy, which mostly rely on predation, digestion and *de novo* synthesis, as opposed to autotrophic symbiotic anemones, in which 60% of carbon flux is provided by symbionts [35]. Numerous studies have characterized glycerol, lipids and amino acids as the major mobile compounds transferred from the symbiont (reviewed in [2], [4]). At the level of metabolism pathways, Phosphoenolpyruvate carboxykinase (PEPCK-C; Kern # 28) had enhanced expression in aposymbiotic conditions. This enzyme catalyzes the rate-controlling step of gluconeogenesis when pyruvate is used as the substrate. Two metabolic pathways can initiate gluconeogenesis: the PEPCK-C dependent pathway, which is fuelled by pyruvate (product of glycolysis), and the PEPCK-C independent pathway, which is fuelled by glycerol [36]. As glycerol is acquired from the symbiont, PEPCK-C is indeed expected to be down-regulated in the symbiotic state.

In zooxanthellate anemones, lipids are the second most abundant transferred mobile compounds from the symbiont [2], [4] therefore requiring up-regulation by the host of several key regulatory enzymes for fatty acid metabolism. Although mobile compounds have been identified using radioactive tracers [2], compound transfer pathways from the symbiont remain to be characterized. Four Kern genes were involved in lipid processing pathways, LSD-2 (lipid storage droplet), SDR12 (terpenoid metabolism), 2-hydroxyacyl-CoA lyase 1 and NPC2. These 4 genes were up-regulated in the symbiotic state (Kern # 2, 13, 15, 17 in Table 1). 2-hydroxyacyl-CoA lyase 1 is responsible for fatty acid alpha-oxidation, a modification specific to fatty acids synthesized from chloroplastic organisms [37]. NPC2 (AvNPC2-D, Kern # 2) is among the most up-regulated genes, substantiating another study which noted that a NPC2 transcript was up-regulated in symbiotic *Aiptasia pulchella* anemones [38]. NPC2 acts in synergy with NPC1 in the transport of sterols (cholesterol) using the late endosomal/lysosomal system [39]–[41]. Noticeably, NPC1 and NPC2 are preferentially expressed in the gastroderm (Table 2). Based on their tissue expression profile and symbiosis-related regulation, NPC1 and NPC2 appear to be major candidate genes for the transport in *A. viridis* of sterol compounds produced by *Symbiodinium*.

Another notable difference between aposymbiotic and symbiotic states is that aposymbiotic-specific Kern genes showed no tissue expression preference. This is in marked opposition to the numerous symbiosis-related Kern genes expressed principally in the gastroderm. Hence, expression of the latter is both limited to the symbiont-containing cells and correlated with the presence of *Symbiodinium*. Although the mechanisms underlying such control are yet to be characterized, several molecular dialogs between host and symbiont can be envisaged.

### Recognition and non-self tolerance by *A. viridis*

The Complement C3 (C3) is a precursor protein involved in adaptive immunity in vertebrates and also in cellular recognition, inflammatory processes and phagocytosis in invertebrates [42], [43]. C3 thus appears central to non-self response in metazoans and homologs have been identified throughout the metazoans, including cnidarians [42], [44]. In the coral *Acropora millepora*, one C3-like protein has been shown to localize in close association with the symbiosome in the gastroderm layer, supporting a role in recognition of *Symbiodinium* [44]. In sea anemones, there are three C3 isoforms in the non-symbiotic *N. vectensis* genome and a minimum of four different C3 isoforms were identified in the *A. viridis* EST dataset (PG and CS, personal communication). We monitored the expression of two of these: AvC3-1 and AvC3-2. Although AvC3-1 expression was mainly restricted to the gastroderm and may well represent the *A. millepora* functional homolog, no differential expression was observed between symbiotic and aposymbiotic anemones (validated by qPCR, Figure 5). On the other hand, AvC3-2 (Kern # 37) was expressed in both tissue layers, but was strongly repressed in the presence of symbionts. The functional divergence between the different C3 isoforms and their relative participation in *Symbiodinium* tolerance remains to be determined. However, based on their respective expression profiles in *A. viridis*, it is conceivable that recognition of and response to *Symbiodinium* may be carried out by different C3 paralogs.

Another recognition process that we uncovered in our experiment is related to the vitamin K-dependent (VKD) γ-carboxylation of Sym32, under the control of the Calumenin protein. In human, Calumenin proteins are composed of 6 to 7 Ca^2+^ binding EF hand domains. They are principally present in the Endoplasmic Reticulum (ER) due to specific ER retention motifs at their COOH termini [26], [45], [46]. Their differences in function are unclear, but they are associated with Ca^2+^ dependent processes, especially with post-translational VKD γ-carboxylation of several proteins [26]. The increase of intracellular vitamin K (a cofactor produced from plant) activates two proteins conserved throughout metazoans: Vitamin K1 2,3-Epoxide Reductase (VKOR) and γ-Carboxylase. The latter recognizes carboxylase recognition sites (CRS) in newly synthesized proteins and adds a CO_2_ group to adjacent glutamic acid (Glu) residues resulting in the production of γ-carboxyglutamic acid (Gla)-containing proteins [47], [48]. VKD proteins have been mainly explored in mammals and include bone marrow proteins such as Osteocalcin, Matrix Gla Protein and Periostin (PN) which are involved in bone formation [49]. The vitamin K cycle is itself under the negative control of Calumenin [26], [46] (Figure 7B). In *A. viridis*, the Calumenin homolog AvCALU-a (Kern # 1) was the most up-regulated gene of the symbiotic state detected in the microarray experiments (Table 1). It is also preferentially expressed in gastrodermal cells. It contains an ER retention motif and is thus expected to down-regulate the VKD γ-carboxylation in the ER of zooxanthellate *A. viridis* cells.

Sym32 was first characterized by Weis and colleagues as a symbiosis-specific protein that is over-represented at the perisymbiotic membrane of the zooxanthellate anemone *A. elegantissima* (AeSym32), but also present at the surface of gastrodermal vesicles in the aposymbiotic state [13], [50]. In *A. viridis*, the Sym32 ortholog (AvSym32, Kern # 4) is up-regulated by more than 40 fold in the symbiotic state and is principally expressed in the gastroderm (Figure 5, Table 1 and Table 2). Both AeSym32 and AvSym32 are composed of two fasciclin I (FasI) domains (Figure 7), functionally associated with cell-cell recognition and thus potentially involved in anemone-zooxanthellae interaction [13], [51]. Importantly, in both sea anemones, the first FasI domain of Sym32 is highly similar in sequence to the first FasI domain of PN, and contains a sequence motif very similar to the CRS domain of PN characterized in vertebrates [49]. Sym32 is thus an implicit substrate for the γ-Carboxylase in sea anemones. As part of the same line of evidence, a 2D immunoblot of zooxanthellate *A. elegantissima* extract hybridized with an anti-Sym32 antibody showed the presence of two spots of 32 KDa, one at PI = 7.9 and the other at PI = 8.2 [51]. Such a PI difference for the same protein could very well correspond to difference between Glu and Gla containing Sym32 proteins since γ-carboxyglutamic acid decreases the PI of a protein (e.g. [52]). However, analysis of freshly isolated *Symbiodinium* (FIZ, where most of the peri-symbiosomal membrane - of host origin - remains attached around the isolated *Symbiodinium* cell) of *A. elegantissima* showed the disappearance of the PI = 7.9 spot in favor of the PI = 8.2 spot [13]. These experiments infer that the Glu-Sym32 protein would localize to the perisymbiotic membrane. Since γ-carboxyglutamic acid residues have been shown to modify the three dimensional structure and Ca^2+^ binding affinity, Glu-Sym32 and Gla-Sym32 would show different ligand properties [53]. Moreover, up-regulation of Calumenin shown in our work suggests an inhibition of γ-carboxylation which would favor the production of Glu-Sym32 in zooxanthellate *A. viridis* cells (Figure 7B). Thus, the vitamin K-dependent γ-carboxylation of Sym32, with its effect on interpartner recognition and the symbiotic process, is definitively a pathway that should be investigated.

### Carbonic anhydrases and the transport of bicarbonate from seawater to the zooxanthellae

The photosynthetic symbionts are separated from the surrounding seawater by several host membranes: membranes of the epidermal cell layer, the collagenous basal membrane, gastrodermal cells, and perisymbiotic vesicles. The main source of inorganic carbon (Ci) for photosynthesis is seawater bicarbonate (HCO_3_^−^), which implies transport of exogenous inorganic carbon through these layers of animal tissue [5], [54]. In seawater (pH 8.2), most inorganic carbon is in the form of HCO_3_^−^, a form that needs carrier-mediation to cross membranes, and that is not readily converted to CO_2_ in the absence of enzymatic action [55], [56]. The currently accepted model for external Ci uptake by the host involves an H^+^-ATPase acidifying the boundary layer where bicarbonate is converted to CO_2_ by an external (likely membrane-bound) carbonic anhydrase isoform [3]. The uncharged CO_2_ molecule then diffuses into the epidermal cell following the concentration gradient created by the extrusion of H^+^ in the external medium. Once in the animal cytoplasm, CO_2_ is equilibrated with HCO_3_^−^ according to the intracellular pH by another CA isoform, which prevents back-diffusion of CO_2_ (for review see [3]). The mechanism of transport of Ci through the other membranes to the symbionts is currently debated (for reviews, see [5], [57], [58]). However, previous works have highlighted the role of a CA localized on the perisymbiotic [59] or algal membrane [60].

According to this model, carbonic anhydrases are crucial enzymes for carbon supply to symbiont photosynthesis. In the present study, the expression of two different carbonic anhydrases (Av_CA2-c and Av_CA2-m) was monitored. Both show highly enhanced expression in symbiotic specimens compared with aposymbiotic ones (4 and 2.9 fold, respectively), suggesting that both isoforms could be involved in the symbiosis/metabolic exchanges between partners. This result is consistent with the relevant work of Weis and her collaborators [6], [59], [61], showing that enzyme activity and transcript quantity are higher in symbiotic than in non-symbiotic specimens of sea anemones *A. pulchella* and *A. elegantissima*.

AvCA2-c isoform, the cytosolic isoform, is equally expressed in both tissue layers. We suggest that AvCA2-c catalyzes the intra-cellular reversible hydration/dehydration of CO_2_ into HCO_3_^−^ to facilitate the transport of CO_2_ through the membranes and cells (Figure 8). AvCA2-m is a membrane-bound isoform specifically expressed in the gastrodermal layer of anemones where symbionts are located. This membrane-bound isoform can be located either on the plasma membrane of the gastrodermal cells, or on the perisymbiotic membrane surrounding the symbiont. In the first case, AvCA2-m would favor the transfer of CO_2_ from one cell layer to another by preventing back-diffusion of CO_2_ through membranes. In the second case, this isoform would catalyze the final conversion of HCO_3_^−^ into CO_2_ for photosynthetic needs. It should be noted that, since in *N. vectensis* at least six different isoforms of carbonic anhydrases have been identified (AM and D. Zoccola, personal communication), other *A. viridis* CA isoforms are expected to contribute in the Ci transport from seawater to *Symbiodinium*.

The previous model of Ci transport assumed that CO_2_ crosses membranes by diffusion through concentration gradients between both sides of the cell plasma membrane. For most of the past century, gas molecules such as CO_2_ were presumed to cross biological membranes merely by diffusing through the lipid phase. This view was challenged recently by studies demonstrating the permeability of water channel aquaporins and certain Rh-family members to CO_2_ [62]–[64]. The physiological function of those channels for CO_2_ transport seems to be particularly important since, for instance, one Rh protein (RhAG) accounts for up to 50% of the CO_2_ transport of human red blood cells [65]. In *A. viridis*, we identified two RhAG isoforms, one of which (Av_RhAG1) is preferentially expressed in the gastroderm of zooxanthellate anemones (Table 2). We suggest that these proteins could have a role within the holobiont to facilitate CO_2_ uptake, possibly in conjunction with the membrane-bound CA2. In human erythrocytes, RhAG and CA2 are part of the same Band 3 multiprotein complex involved in anion exchanges [66], [67]. It is worth mentioning that a role of Av_RhAG1 in NH_4_ transport is equally valid, as in the cnidarian-dinoflagellate symbiosis, ammonium resulting from host metabolism is not excreted into the surrounding water but is immediately re-assimilated by the algae, either through diffusion or by a transporter, and then recycled [4], [68].

### Is the combination of gene duplication and gastroderm-specific expression a symbiosis adaptation by anthozoans?

Within the set of Kern genes up-regulated in the symbiotic condition, we noticed that some of the genes with a proposed function in symbiosis, including Sym32, Calumenin, and NPC2, were expressed as 2 or more related copies. Phylogenetic analysis allowed the conclusion to be made that there were cnidarian-specific gene duplications. Remarkably, our expression results in *A. viridis* showed that these cnidarian-specific duplicates were both preferentially expressed in the gastroderm (hosting zooxanthellae) and in the symbiotic condition.

Such cnidarian-specific gene duplications could correlate with the amenability of various cnidarians to have accepted photosynthetic endosymbionts during evolution. However, these gene duplications are not restricted to symbiotic anemones (e.g. *N. vectensis*). Their selective advantage with regard to symbiosis therefore remains to be determined, together with the origin of endosymbiosis in cnidarians: was photosynthetic symbiosis acquired or lost in various branches of the phylum? Moreover, at the transcriptional level, we have shown for several cases that expression of one isoform among the duplicated gene copies was specifically up-regulated both in the gastroderm and in the presence of symbionts. Thus, we suggest that these neofunctionalizations would be associated with the physiological constraints of endosymbiosis and would be tuned to the presence of zooxanthellae by transcriptional control. It is not known how such control is exerted or whether such gene regulation is restricted to symbiotic cnidarians. Comparative tissue expression of the different orthologs in non-symbiotic cnidarians, such as *N. vectensis*, would provide insight into the adaptive origin of symbiosis.

### Coral bleaching and transcription plasticity

One of our most unexpected results was that outside the subset of Kern genes, many differentially expressed genes were up- or down-regulated in only few individual anemones, despite the fact that anemones were in a fixed symbiotic or aposymbiotic state before sampling. Similar inter-individual variable expression profiles have already been quoted in two studies comparing the response of *A. millepora* corals to environmental changes [15], [69].

On the other hand, several groups studying symbiosis breakdown in diverse cnidarian species showed that different cellular mechanisms were observed during loss of zooxanthellae, including apoptosis, necrosis, exocytosis, or phagocytosis [11]. Such variability in the cellular processes involved may reflect distinct causes of bleaching, i.e. different responses to variable environmental changes. Indeed, bleaching can be caused by a multitude of environmental stressors including changes in seawater temperature, salinity, ultraviolet radiation, increased sedimentation, nutrients and pollutants [70]. In response to different stresses, the host-associated microbiota is greatly and specifically modified within the whole holobiont (i.e. the community composed of cnidarian host, dinoflagellates and associated microbes) [71].

In the case of the Mediterranean symbiotic cnidarian used here, *Symbiodinium* belongs to the same clade temperate A. However, our results on the gene copy numbers of only 3 genes (EF2, SPS and APX) showed unexpected high polymorphism in the *Symbiodinium* hosted by anemones collected from neighboring location. In addition, we showed that the abundance of zooxanthellae per host cell is variable within our set of symbiotic sea anemones. Thus, variation in the associated microbiota or in *Symbiodinium* sub-clades may have stable extended effects on the host gene expression profile. This underlines that, although the term “bleached” defines one visual phenotype resulting from various responses, it can result from diverse expression profiles, and probably from various conditions of aposymbiosis. Indeed, not all bleached cnidarians die.

*A. viridis* can be sampled at different time points without harm to the anemone, and is therefore an excellent model organism for kinetics experiments. Additionally, since sea anemones are non-calcifying anthozoans, they allow the study of symbiosis-associated processes outside the cross-regulatory pathways of mineralization found in corals. It would be of interest, using the same array technique, to monitor the kinetics of the specific gene expression response to one or combination of different environmental stressors. In addition to giving a transcriptional map of the gene-specific response to stress, it would highlight whether different bleaching expression profiles are fixed in time or whether they tend to stabilize at a unique Kern profile after an adaptation period. Moreover, the unique amenability of *A. viridis* to separate the ectoderm from the endoderm will permit us to partition the scope of the environmental response in the cellular layer hosting the symbionts (gastroderm) and in the tissue in direct contact to the milieu (epidermis).

## Materials and Methods

### Collection and maintenance of *A. viridis* specimens

Mediterranean sea anemone specimens, *Anemonia viridis* (Forskål, 1775), were collected in five locations on and around the French Riviera (Figure S2): Antibes (Salis and Croutons sites), Villefranche-sur-Mer, Monaco and Menton. A total of 5 symbiotic and 6 aposymbiotic specimens were used in this study.

Symbiotic specimens Sy1 and Sy3–5 were collected from Antibes Croutons (Figure S2), Sy2 was collected from Antibes Salis. These anemones were maintained for several months in seawater aquaria at 17.0±0.5°C with weekly water renewal. A metal halide lamp (HQI-TS 400 W, Philips) provided light at a constant saturating irradiance of 250 µmol m^−2^ s^−1^ on a 12/12 h light/dark cycle. Naturally-occurring aposymbiotic animals were sampled from the public aquaria of the Oceanographic Museum of Monaco and originated from different sites in Monaco (AS1 and AS2) or Menton (AS3). The three other aposymbiotic specimens were obtained after either a thermal stress-induced bleaching (AS4, AS5, collected in Antibes-Crouton) or a treatment with the catalase inhibitor aminotriazol (AS6, collected in Villefranche-sur-Mer, [7]). These latter 3 anemones were maintained in the dark (necessary to keep them bleached) for a minimum of 2 months. Both symbiotic and stress induced aposymbiotic anemones were fed twice a week with frozen *Artemia salina*.

Sy3, Sy4 and Sy5 specimens were later subjected to an 8°C-temperature increase (17 to 25°C) to assess imposed stress response. Tentacles were sampled over a 48 h kinetic period (t0, t24h and t48h) and then subjected to RT-qPCR experiments.

Specimen sampling was always at 10:00 (2 hours after light start) to avoid circadian effect of gene expression fluctuations.

### Nucleic acid extraction

#### RNA extraction

Total RNA was extracted from whole tentacle samples (epidermis plus gastroderm and zooxanthellae) or separated tissues using Trizol Reagent (Invitrogen), as described previously [20]. Gastrodermal (gastroderm and zooxanthellae) and epidermis layers were separated as described previously [9]. A DNase treatment (RQ1-DNAse, Promega) was performed on the RNA sample to avoid genomic DNA contamination.

#### gDNA extraction

Genomic DNA was extracted as follows: samples (whole tentacles, separated tissue layers (epidermis or gastroderm) or cultured zooxanthellae) were first immersed in [100 mM Tris pH = 8.2; 100 mM EDTA; 500 mM NaCl; 2.5% triton] to block DNases, and then transferred into [4 M Thiocyanante Guanidium; 25 mM Sodium citrate; 1% Triton X100; 50 µg/ml Proteinase K; 5 mM EDTA; 50 mM DTT] before potter homogenization. Homogenates were incubated at 55°C for 1 hour. Genomic DNA was purified by phenol/chloroform/isoamyl alcohol extraction and isopropanol precipitation.

### *A. viridis* oligoarray hybridizations

Over 2,000 genes were selected from the *A. viridis* clustered and annotated EST collection [20] for their putative participation in symbiotic processes. Genes were selected by keyword search in their functional annotation for matches with the following terms: intracellular transport, metabolic processes (lipids, proteins), signal transduction, organelle organisation and biogenesis, response to stress, trans-membrane, apoptosis and cell death. Two thousand 60-mer oligonucleotides were designed (Eurogentec, Belgium) and printed on slides in triplicate (Eurogentec, Belgium). A luciferase oligonucleotide was also spotted as an external control. This *A. viridis* Oligo2K version 1.0 oligoarray is fully described under platform record GPL10546, stored in the Gene Expression Omnibus (GEO) at NCBI (http://www.ncbi.nlm.nih.gov/geo).

cDNA synthesis and labelling was performed from total RNA using the ChipShot Direct labelling and Clean-up system (Promega), according to the manufacturer's instructions. Five ng of a Lux mRNA exogenous standard was added to each mRNA sample before labelling. RNA quality was evaluated using the Agilent Bioanalyzer 2100 and quantified on a ND-1000 Spectrophotometer (NanoDrop). Then, 500 ng of each labeled sample were mixed in Hi-RPM hybridization buffer (Agilent), and incubated on the array overnight at 47°C. Slides were scanned after post-hybridization washes, using a GenePix 4200A scanner (Axon Instruments). Data acquisition and quality control were performed using Genepix Pro software.

### Experimental design and statistical analyses

Dye-swap experiments were performed for each hybridization condition. Two different sets of hybridizations were performed (Figure S3): i) symbiotic *versus* aposymbiotic specimens and ii) epidermis *versus* gastroderm tissues. The same AS6 extract was used as the sample reference in all experiments. Experimental data and associated microarray designs were deposited in the National Center for Biotechnology Information (NCBI) Gene Expression Omnibus (GEO) (http://www.ncbi.nlm.nih.gov/geo/) under SuperSeries record: GSE22375 and platform record GPL10546.

Background (positive offset of 50) was evaluated according as described in [72]. The data were normalized by the print-tip loess method (within-array normalization) and by quantile method (between-array normalization) using the LimmaGUI package from Bioconductor [23]. Means of ratios from all comparisons were calculated for each gene, and B test analysis was done using LimmaGUI. Differential gene expression was determined by the Bayesian statistical method (B value), with a cut-off of zero for the B value and a log ratio |M|>0.590 as significant. All normalized data sets were registered in the GEO database under the accession number GSE22375.

Cluster 3.0 software [73] was used to estimate the hierarchical clustering between individual anemone array results. The following parameters (complete, average, and centroid linkage) were tested and gave similar results.

### Real-time quantitative PCR experiments

Specific primers amplifying around 100 bp were designed using the software Primer3 [74]. The primer sequences used in this study are listed in Table S3. Amplicon specificity for either *A. viridis* or *Symbiodinium* was tested against epidermal (extracts without zooxanthellae) or CZ (zooxanthellae culture) extracts, respectively. Expected length of the amplicons was checked by agarose gel electrophoresis after regular PCR amplification. Primer efficiencies were determined using standard curve analysis with a 10-fold dilution series of pooled cDNA from both control and treated samples (data not shown), and ranged from 1.8 to 2. The qPCR products were sequenced (Macrogen Inc, Korea) and all matched the expected product identities.

cDNAs were prepared using SuperScriptII reverse transcriptase (Invitrogen) and a mixture of oligodT and random primers, according to the manufacturer's instructions. Transcript level quantification was performed using the SYBR green fluorescence method and a Light Cycler 480 (Roche). The PCR conditions were as follows: 1× SYBR green mix (LC480 SYBR Green Master Mix, Roche), 100 nM primers and 2.5 ng of cDNA in a total volume of 15 µl. Each sample was run in triplicate using the following PCR parameters: 94°C for 10 min, followed by 40 cycles of 15 s at 94°C, 20 s at 60°C and 15 s at 72°C, then a dissociation curve step (60 to 95°C) to confirm the absence of non-specific products. The dissociation curves showed a single amplification product and no primer dimers.

Several control genes were chosen based on the microarray results as a whole (most stably expressed genes, in all tested conditions), and the expression stability of ten putative control genes was evaluated using the GeNorm software [75]. A reliable normalization factor was calculated based on the expression level of the most stable control genes. The control genes finally selected in this study are RPLP0, RCC2, and COP-γ. Expression levels of target genes were normalized using the normalization factor described above and the results given as expression relative to the aposymbiotic specimen (AS6) value as calibrator (reference). The significance of the results was tested using *t*-tests (software Jump 5.1, Cary, USA). Results were considered statistically significant when P<0.05.

### Bleaching quantification

The relative abundance of zooxanthellae in *A. viridis* cells was quantified using a real-time quantitative qPCR method. For each organism, three nuclear genes were chosen from available datasets: COP-γ (Coatomer subunit gamma), RCC2 (Regulator of Chromosome Condensation protein 2) and NPC1 (Niemann Pick type C1) for *A. viridis*, and EF2 (Elongation factor 2), Sucrose phosphate synthase (SPS) and Ascorbate peroxidase (APX) for *Symbiodinium* clade temperate A. Primer design was performed using Primer3 and the same parameters described above; the primer sequences are given in the Table S3. Standard curves were generated with six logarithm dilutions of corresponding cloned sequences. Results are expressed as relative quantification of *Symbiodinium* nuclei (or nuclear genes) to *A.viridis* nuclei.

### Sequence analyses

Sequencing of either *A.viridis* library clones or newly cloned cDNAs was performed by Macrogen Inc.

Signal peptides and Trans-membrane domains were predicted using SignalP and TMHMM, respectively, from the CBS prediction servers (www.cbs.dtu.dk/services). GPI anchors were predicted with PredGPI (http://gpcr.biocomp.unibo.it/predgpi). Other Domains were inferred from PFAM (pfam.sanger.ac.uk). Blast analysis tools used were from NCBI (blast.ncbi.nlm.nih.gov).

Functionnal annotation of the *A. viridis* EST dataset (GO annotations) has been performed using Blast2GO [20]. Statistical assessment of annotation differences between SY-genes (test group) and APO-genes (reference group) was performed using the Gossip package [76], that employs Fisher's Exact Test to estimate the significance of associations between two categorical variables.

Sequence alignments were performed using MultAlin [77] and ClustalW with Blosum62 default parameters. Alignments were optimized manually using the 2 computer generated alignments as a model. Using the segment of the sequence alignment conserved in all sequences (bordered by the bar above alignments in Figures S5, S6, S7), the best-fitted substitution model was evaluated using ProtTest [78]. Using parameters indicated in the Figure legend of each alignment tree, a Maximum Likelihood tree was determined using phyML and branches support were calculated using aLRT [79]. Alternatively, Bayesian analysis using MrBayes 3-1.2 (mrbayes.csit.fsu.edu) was conducted with the following settings: fixed rate amino acid model was set to mixed (prset aamodelpr = mixed) and proportion of invariable sites model was combined with the Gamma model (lset rates = invgamma).

## Supporting Information

Figure S1 The sea anemone model *Anemonia viridis* and the symbiosis-dedicated oligoarray. A. Schematic section through an *A. viridis* polyp, showing the two tissues (compartments) composing the animal: the epidermis (“E”) and the gastroderm hosting the photosynthetic zooxanthellae (“G”). B. The 2,000 genes compiled on the oligoarray were selected from an *A. viridis* clustered and annotated EST dataset according to putative participation in symbiotic processes. Genes were classified by GO terms according to Molecular Function, Biological Process and Cellular Components. Histogram values are given as the percentage of total within each GO category. C. Significant GO terms enrichment between the 136 annotated genes identified as up-regulated in the symbiotic state (SY genes; test group) and up-regulated in the aposymbiotic state (APO genes; reference group). Statistical analysis was conducted using Gossip package which employs Fisher's Exact Test (p value<0.05).
(TIF)

Figure S2 Sampling areas. Map of the French Riviera coastal area, showing the different diving locations where sea anemones were collected. Below stands the name of the 11 *A. viridis* anemones used in this study and their collection location, as well as the stress which lead AS1–6 anemones to bleach.
(TIF)

Figure S3 Counting of relative host to symbiont nuclear ratio in individual specimen using real time PCR. Total genomic DNA was extracted from the 11 sea anemones tentacles (Sy1–5 and AS1–6), a dissected epidermal tissue (Ep) and culture *Symbiodinium* (CZ). The gDNAs were used as template for real-time quantitative PCR with primers specific for the *Symbiodinium* EF2, APX and SPS genes and the *A. viridis* COP-γ, RCC2 and NPC1 genes. A. Comparison of the relative *A.viridis* gene loci number (top panel) shows that most gene ratios are around 1∶1 in the different individuals whereas relative *Symbiodinium* gene loci number (bottom panel) shows variable numbers, essentially due to variation in the EF2 loci number. B. Comparative gene ratio between *Symbiodinium* SPS, APX and EF2 versus *A.viridis* RCC2 (top panel) and NPC1 (bottom panel). Both histograms confirm the symbiotic and aposymbiotic state of the specimen used in this study, individual showing similar pattern than in Figure 1.
(TIF)

Figure S4 Schematic diagrams of microarray experimental design. A. cDNAs from symbiotic anemones Sy1–Sy5 and aposymbiotic anemones AS1–AS5 were hybridized against the same cDNA sample from the AS6 aposymbiotic sea anemone. Dye-swap hybridizations were performed for all experimental conditions. B. cDNAs from E (epidermis) and G (gastroderm+zooxanthellae) tissue fractions from 3 different anemones (Sy3–Sy5) were hybridized against each other or against the AS6 (C+D) reference sample. Dye-swap hybridizations were performed for all experimental combinations.
(TIF)

Figure S5 Phylogenetic analysis of the Calumenin gene family. Human proteins belonging to the Calumenin protein family (as defined in [26]) [Hs_ret1 (NP_002892.1), Hs_ret3 (NP_065701.2), Hs_Cab45-G (AAH06211.1), Hs_CALU (AAC17216.1), ERC-55-E (NM_002902)], their homologs from *Ciona intestinalis* [CiCALUa (NP_001027627), CiCALUb (XP_002123414) and CiCAB45 (XP_002121909)], *Strongylocentrotus purpuratus* [SpCALUa (XP_001179199), SpCALUb (XP_797927) and SpCAB45 (XP_783813)], *Tribolium castaneum* [TcCALUa (XP_974976), TcCALUb (XP_970591) and TcCAB45 (XP_969624)], *Drosophila melanogaster* [DmCALUa (NP_477392), DmCALUb (NP_608899) and DmCAB45 (NP_732406)], *Caenorhabditis elegans* [CeCALUa (NP_001024806), CeCALUb (NP_491936) and CeCAB45 (NP_495338)], *Trichoplax adherans* [TaCALUa (XP_002109885), TaCALUb (XP_002109316) and TaCALUc (XP_002118306)], *Nematostella vectensis* [Nv_CALUa (jgi|Nemve1|174458|), Nv_CALUb (jgi|Nemve1|86027|(extended)), Nv_CALUc (jgi|Nemve1|138173|(extended)), Nv_CALUd (jgi|Nemve1|190767|), Nv_CALUe (jgi|Nemve1|184260|(extended)), Nv_CALUf jgi|Nemve1|79348|), Nv_CALUg (jgi|Nemve1|235717|(corrected_JGI_CAGH9785)), Nv_CALUh (jgi|Nemve1|248118|), Nv_Cab45 (jgi|Nemve1|102868|(corrected_JGI_CAGH7712))] and those identified in *A. viridis* [AvCALUa (Av01015l05r1), AvCALUb (CL1014ct1), AvCALUc (CL101ct1)] were aligned using MultAlin and ClustalW. Signal peptides (yellow highlight) were predicted using SignalP (*Emanuelsson O, et al., Nature Protocols, 2007*). Bayesian phylogenetic tree was calculated using MrBayes 3-1.2.
(TIF)

Figure S6 Phylogenetic analysis of NPC2. Human NPC2 (HsNPC2, NP_006423), and homologs from *Danio rerio* (DrNPC2a, NP_001122191& DrNPC2b, NP_775331) *Ciona intestinalis* (CiNPC2a, XP_002121795 & CiNPC2b, XP_002127695), *Strongylocentrotus purpuratus* (SpNPC2, XP_784998), *Caenorhabditis elegans* (CeNPC2, NP_497671), *N. vectensis* (NvNPC2a, XP_001627355 & NvNPC2b, XP_001622874), *A. viridis* (AvNPC2a, CL214Ct1 & AvNPC2d, CL1319Ct1), *Trichoplax adherans* (TaNPC2,XP_002109765), *Saccharomyces cerevisiae* (*S.cerevisiae*, Q12408) and *Schizosaccharomyces pombe* (*S.pombe*, Q9C0X9) were aligned with MultAlin and ClustalW. Bayesian phylogenetic tree was calculated using MrBayes 3-1.2.
(TIF)

Figure S7 Phylogenetic analysis of the Sym32/Periostin/BGH3 gene family. A. Sea anemone FasI-containing proteins. Protein sequences for *A. elegantissima* Sym32 (Aesym32; AAF65308), *A. viridis* Sym32 (AvSym32; CL363Contig1) and Periostin (AvPN; Rav02077g18) and *N. vectensis* 2Sym (Nv2Sym; misassembeled [see jgi ESTcluster 2667343_11] and corrected from XP_001629263 and XP_001629262) and Periostin (NvPN; extended from jgi|Nemve1|238669|estExt_fgenesh1_pg.C_70144) were aligned using MultAlin. Signal peptides (yellow highlight) were predicted using SignalP. B. Individual FasI domain alignment and phylogenetic relationships. All the different FasI domains from Human Periostin (HsPN1-4; Q15063) and BIGH3 (Hs_BGH31-4; Q15582) were aligned with those of the sea anemone homologs using MultAlin. The CRS (Carboxylase Recognition Site, as described in [49]) of the HsPN-1 and HsBIGH3-1 first FasI domains is highlighted. Using the segment of the sequence alignment conserved in all sequences, the best-fitted substitution model was evaluated using ProtTest. Using parameter [LG+G; gamma = 1.755], a Maximum Likelihood tree was determined using phyML. Bayesian phylogenetic tree was calculated using MrBayes 3-1.2.
(TIF)

Table S1 List of the genes up-regulated in the Symbiotic condition. Legend as in Table 2.
(XLS)

Table S2 List of the genes up-regulated in the Aposymbiotic condition. Legend as in Table 2.
(XLS)

Table S3 Primer sequences used in this article.
(XLS)

Table S4 Gene sequences used in this article.
(XLS)
