# Supplementary figures and images for: Adaptations to Endosymbiosis in a Cnidarian-Dinoflagellate Association: Differential Gene Expression and Specific Gene Duplications
Source: PLoS Genet. 2011 Jul 21;7(7):e1002187. doi: 10.1371/journal.pgen.1002187 (PMC3141003; doi:10.1371/journal.pgen.1002187)

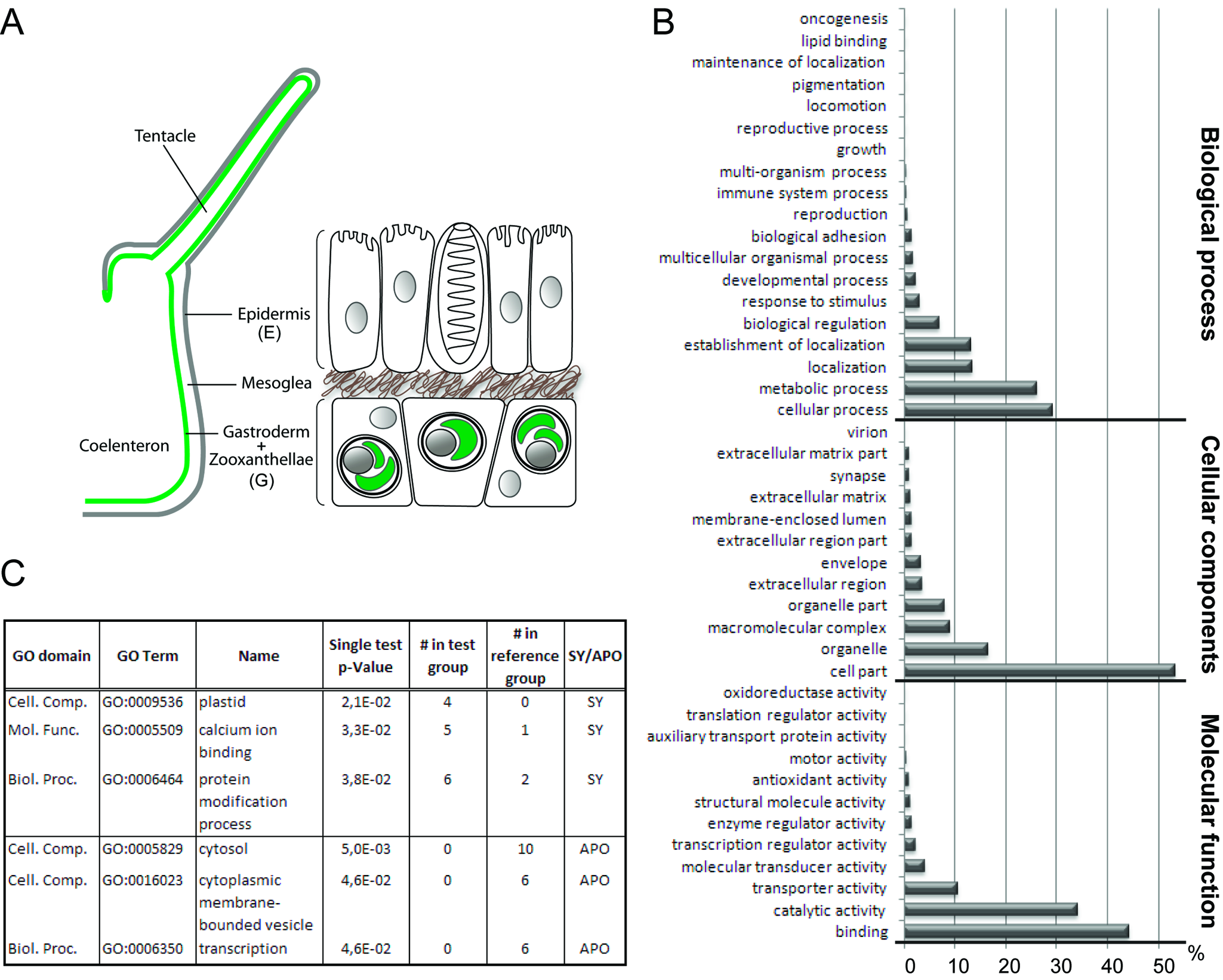

Supplement: Figure S1 — The sea anemone model Anemonia viridis and the symbiosis-dedicated oligoarray. A. Schematic section through an A. viridis polyp, showing the two tissues (compartments) composing the animal: the epidermis (“E”) and the gastroderm hosting the photosynthetic zooxanthellae (“G”). B. The 2,000 genes compiled on the oligoarray were selected from an A. viridis clustered and annotated EST dataset according to putative participation in symbiotic processes. Genes were classified by GO terms according to Molecular Function, Biological Process and Cellular Components. Histogram values are given as the percentage of total within each GO category. C. Significant GO terms enrichment between the 136 annotated genes identified as up-regulated in the symbiotic state (SY genes; test group) and up-regulated in the aposymbiotic state (APO genes; reference group). Statistical analysis was conducted using Gossip package which employs Fisher's Exact Test (p value<0.05). (TIF) [file pgen.1002187.s001.tif]

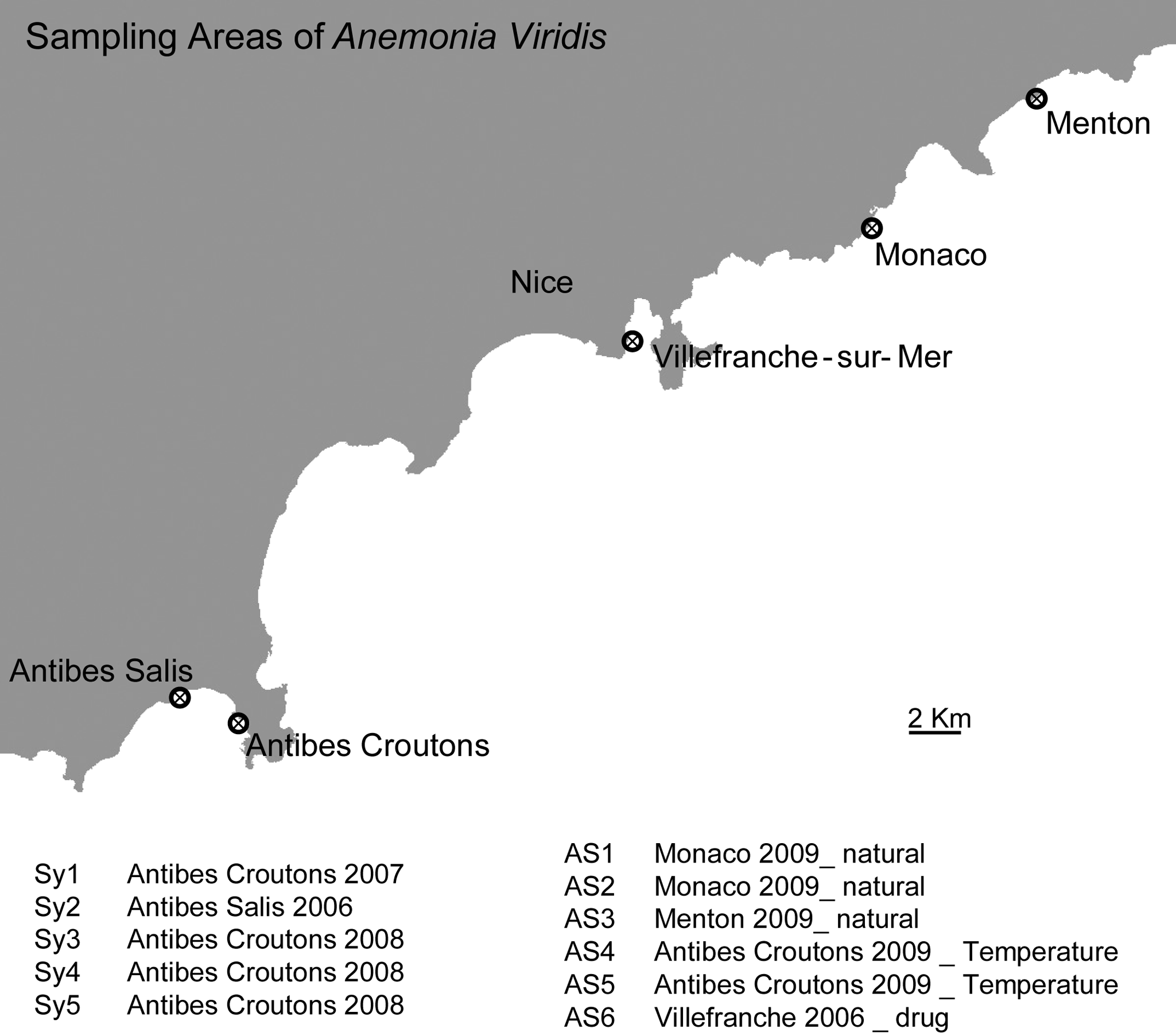

Supplement: Figure S2 — Sampling areas. Map of the French Riviera coastal area, showing the different diving locations where sea anemones were collected. Below stands the name of the 11 A. viridis anemones used in this study and their collection location, as well as the stress which lead AS1–6 anemones to bleach. (TIF) [file pgen.1002187.s002.tif]

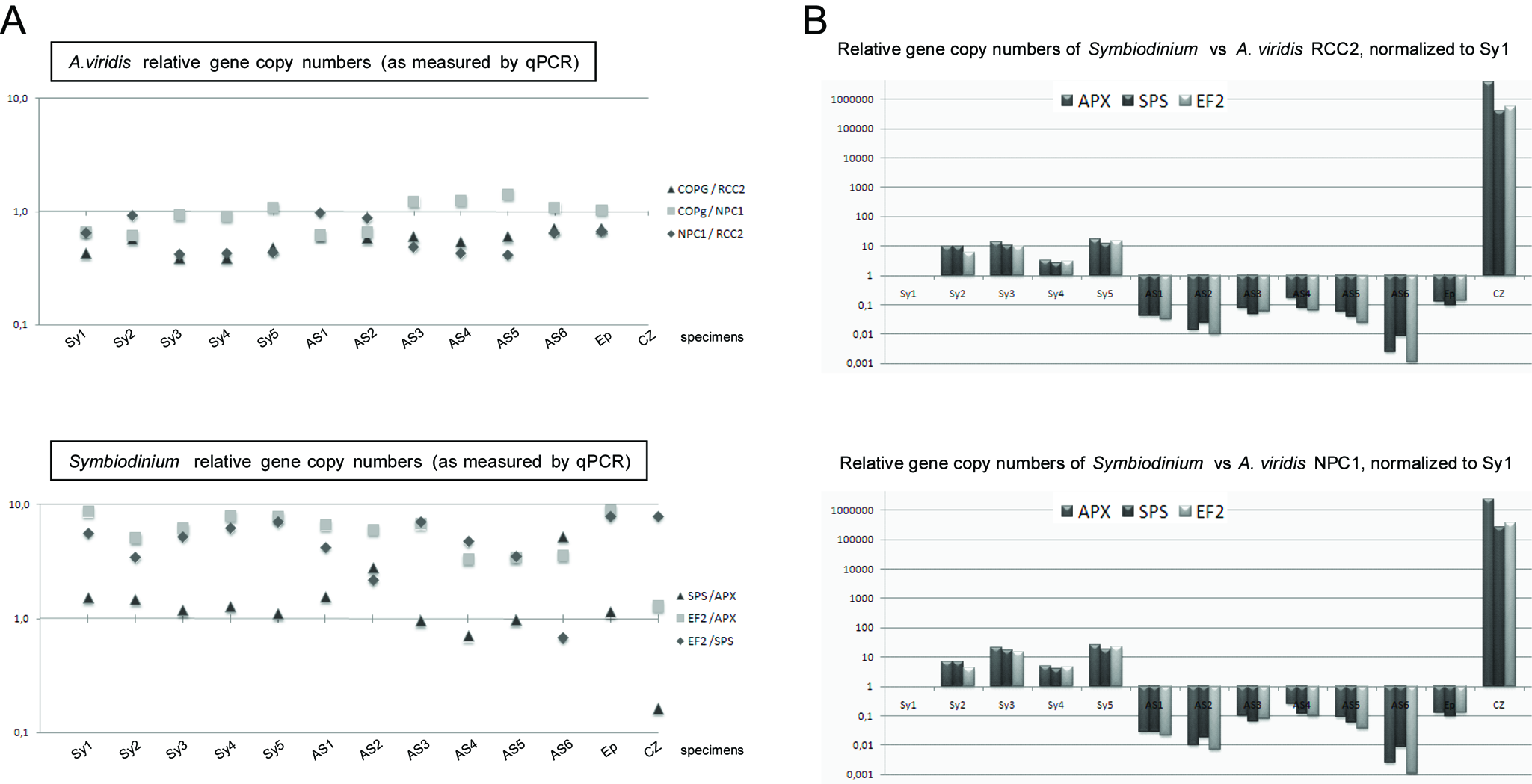

Supplement: Figure S3 — Counting of relative host to symbiont nuclear ratio in individual specimen using real time PCR. Total genomic DNA was extracted from the 11 sea anemones tentacles (Sy1–5 and AS1–6), a dissected epidermal tissue (Ep) and culture Symbiodinium (CZ). The gDNAs were used as template for real-time quantitative PCR with primers specific for the Symbiodinium EF2, APX and SPS genes and the A. viridis COP-γ, RCC2 and NPC1 genes. A. Comparison of the relative A.viridis gene loci number (top panel) shows that most gene ratios are around 1∶1 in the different individuals whereas relative Symbiodinium gene loci number (bottom panel) shows variable numbers, essentially due to variation in the EF2 loci number. B. Comparative gene ratio between Symbiodinium SPS, APX and EF2 versus A.viridis RCC2 (top panel) and NPC1 (bottom panel). Both histograms confirm the symbiotic and aposymbiotic state of the specimen used in this study, individual showing similar pattern than in Figure 1. (TIF) [file pgen.1002187.s003.tif]

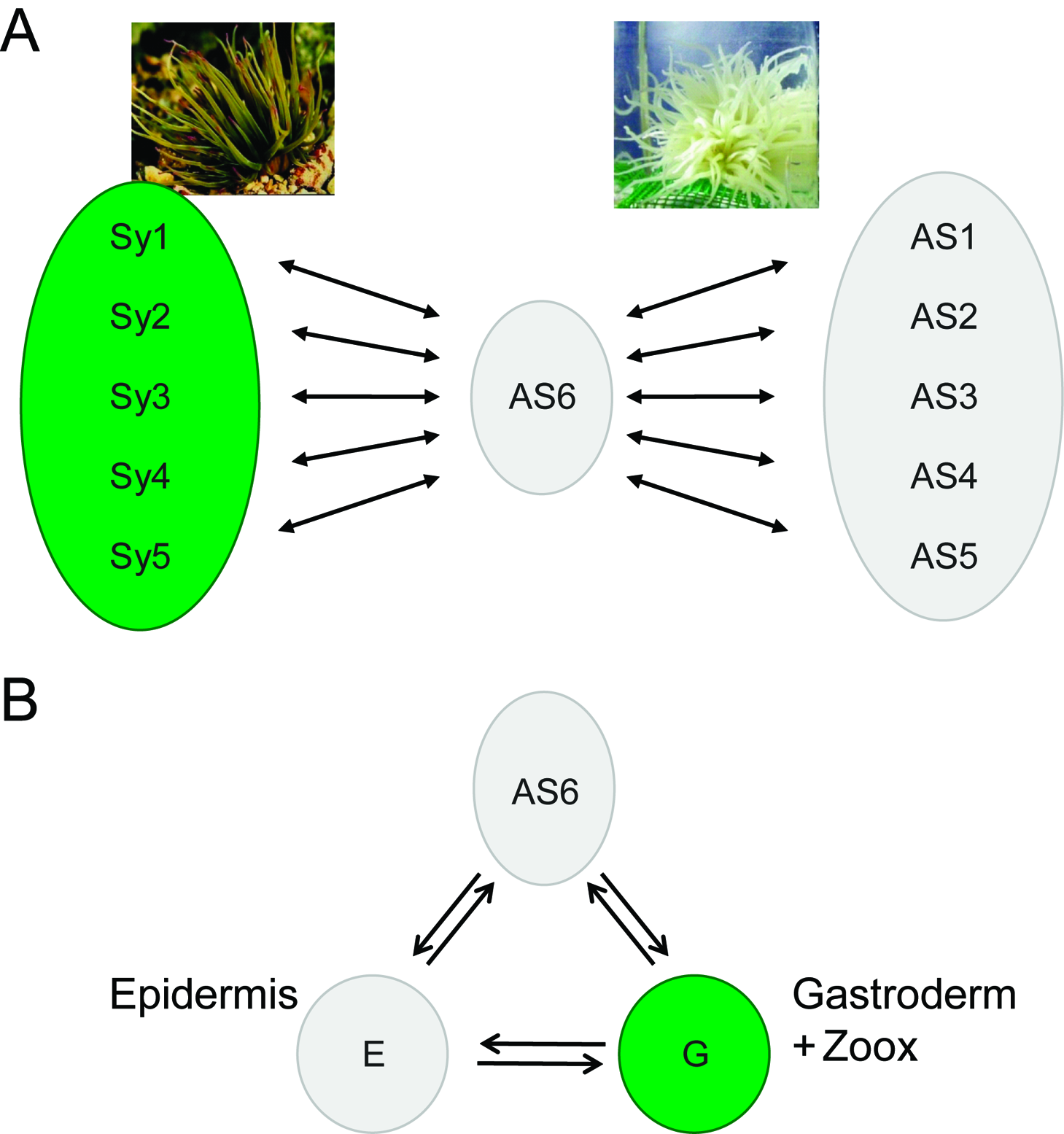

Supplement: Figure S4 — Schematic diagrams of microarray experimental design. A. cDNAs from symbiotic anemones Sy1–Sy5 and aposymbiotic anemones AS1–AS5 were hybridized against the same cDNA sample from the AS6 aposymbiotic sea anemone. Dye-swap hybridizations were performed for all experimental conditions. B. cDNAs from E (epidermis) and G (gastroderm+zooxanthellae) tissue fractions from 3 different anemones (Sy3–Sy5) were hybridized against each other or against the AS6 (C+D) reference sample. Dye-swap hybridizations were performed for all experimental combinations. (TIF) [file pgen.1002187.s004.tif]

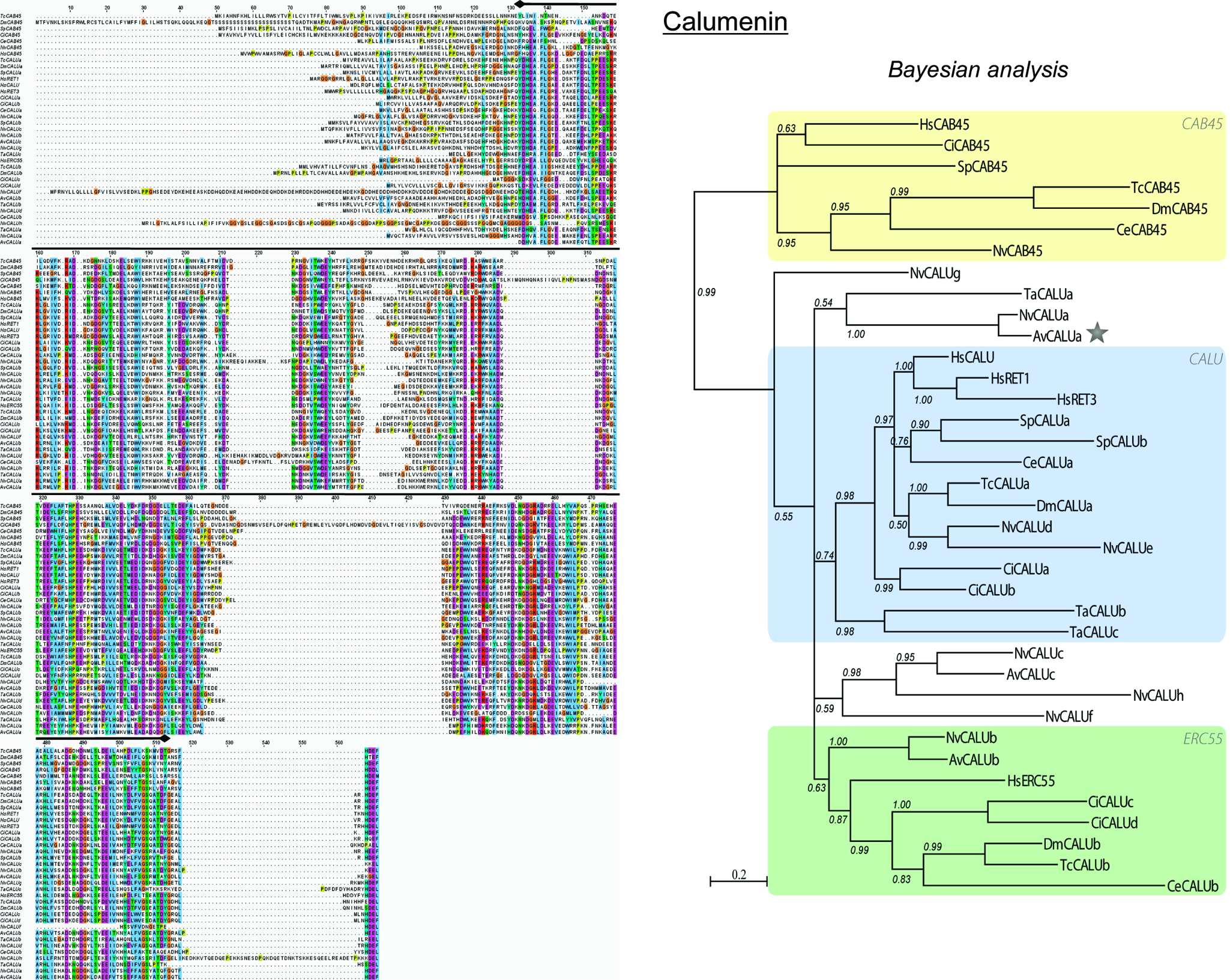

Supplement: Figure S5 — Phylogenetic analysis of the Calumenin gene family. Human proteins belonging to the Calumenin protein family (as defined in [26]) [Hs_ret1 (NP_002892.1), Hs_ret3 (NP_065701.2), Hs_Cab45-G (AAH06211.1), Hs_CALU (AAC17216.1), ERC-55-E (NM_002902)], their homologs from Ciona intestinalis [CiCALUa (NP_001027627), CiCALUb (XP_002123414) and CiCAB45 (XP_002121909)], Strongylocentrotus purpuratus [SpCALUa (XP_001179199), SpCALUb (XP_797927) and SpCAB45 (XP_783813)], Tribolium castaneum [TcCALUa (XP_974976), TcCALUb (XP_970591) and TcCAB45 (XP_969624)], Drosophila melanogaster [DmCALUa (NP_477392), DmCALUb (NP_608899) and DmCAB45 (NP_732406)], Caenorhabditis elegans [CeCALUa (NP_001024806), CeCALUb (NP_491936) and CeCAB45 (NP_495338)], Trichoplax adherans [TaCALUa (XP_002109885), TaCALUb (XP_002109316) and TaCALUc (XP_002118306)], Nematostella vectensis [Nv_CALUa (jgi|Nemve1|174458|), Nv_CALUb (jgi|Nemve1|86027|(extended)), Nv_CALUc (jgi|Nemve1|138173|(extended)), Nv_CALUd (jgi|Nemve1|190767|), Nv_CALUe (jgi|Nemve1|184260|(extended)), Nv_CALUf jgi|Nemve1|79348|), Nv_CALUg (jgi|Nemve1|235717|(corrected_JGI_CAGH9785)), Nv_CALUh (jgi|Nemve1|248118|), Nv_Cab45 (jgi|Nemve1|102868|(corrected_JGI_CAGH7712))] and those identified in A. viridis [AvCALUa (Av01015l05r1), AvCALUb (CL1014ct1), AvCALUc (CL101ct1)] were aligned using MultAlin and ClustalW. Signal peptides (yellow highlight) were predicted using SignalP (Emanuelsson O, et al., Nature Protocols, 2007). Bayesian phylogenetic tree was calculated using MrBayes 3-1.2. (TIF) [file pgen.1002187.s005.tif]

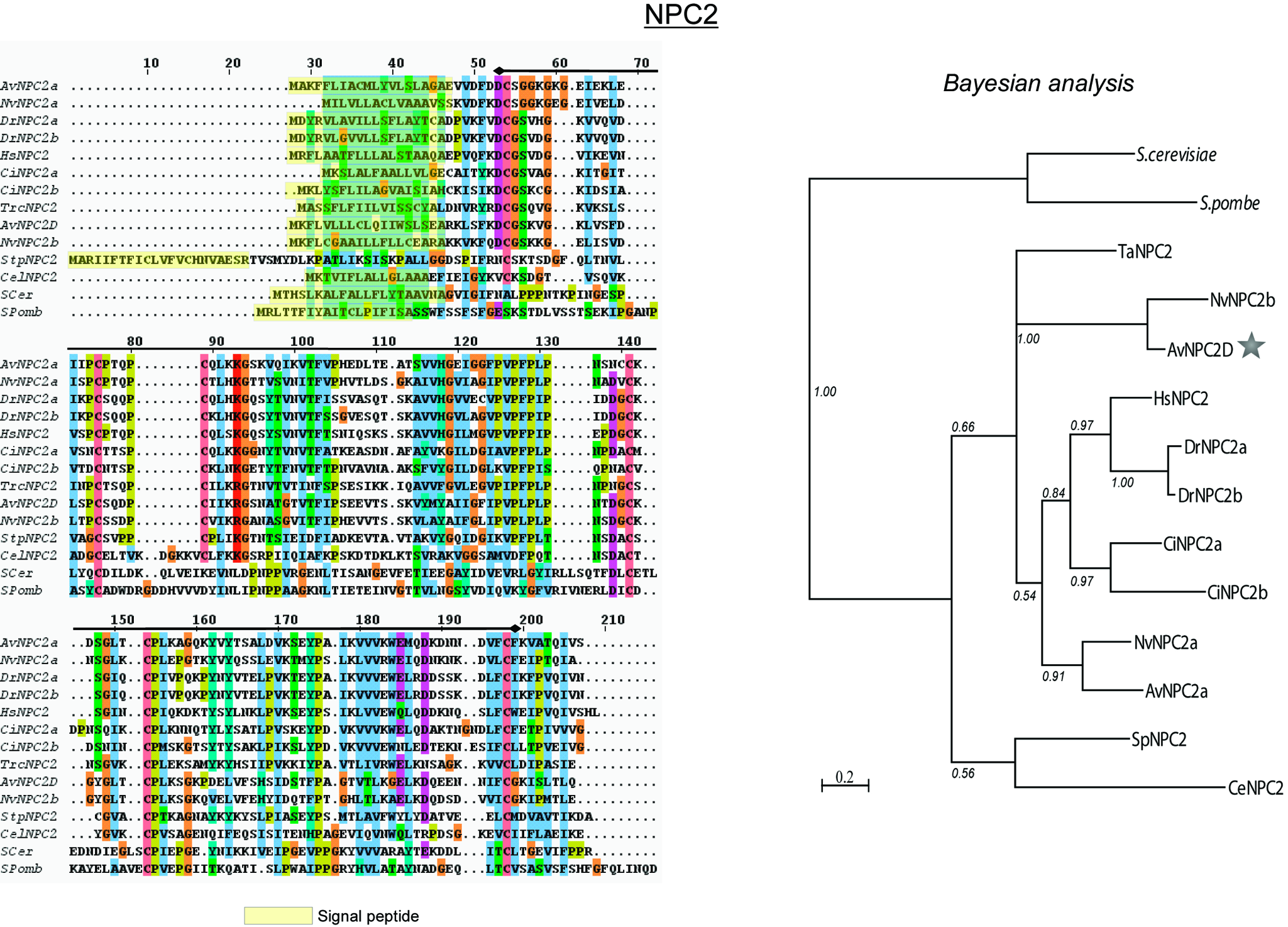

Supplement: Figure S6 — Phylogenetic analysis of NPC2. Human NPC2 (HsNPC2, NP_006423), and homologs from Danio rerio (DrNPC2a, NP_001122191& DrNPC2b, NP_775331) Ciona intestinalis (CiNPC2a, XP_002121795 & CiNPC2b, XP_002127695), Strongylocentrotus purpuratus (SpNPC2, XP_784998), Caenorhabditis elegans (CeNPC2, NP_497671), N. vectensis (NvNPC2a, XP_001627355 & NvNPC2b, XP_001622874), A. viridis (AvNPC2a, CL214Ct1 & AvNPC2d, CL1319Ct1), Trichoplax adherans (TaNPC2,XP_002109765), Saccharomyces cerevisiae (S.cerevisiae, Q12408) and Schizosaccharomyces pombe (S.pombe, Q9C0X9) were aligned with MultAlin and ClustalW. Bayesian phylogenetic tree was calculated using MrBayes 3-1.2. (TIF) [file pgen.1002187.s006.tif]

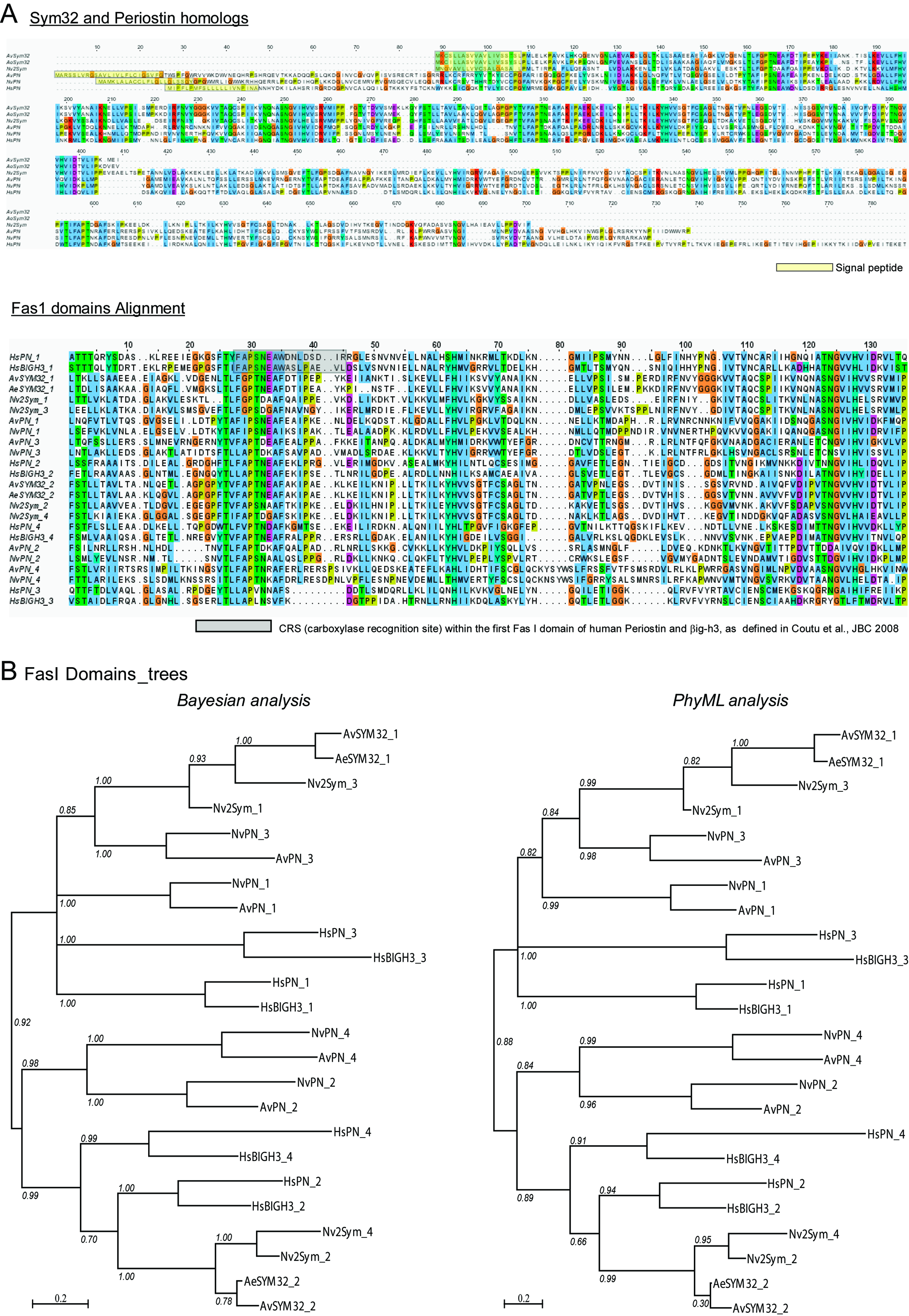

Supplement: Figure S7 — Phylogenetic analysis of the Sym32/Periostin/BGH3 gene family. A. Sea anemone FasI-containing proteins. Protein sequences for A. elegantissima Sym32 (Aesym32; AAF65308), A. viridis Sym32 (AvSym32; CL363Contig1) and Periostin (AvPN; Rav02077g18) and N. vectensis 2Sym (Nv2Sym; misassembeled [see jgi ESTcluster 2667343_11] and corrected from XP_001629263 and XP_001629262) and Periostin (NvPN; extended from jgi|Nemve1|238669|estExt_fgenesh1_pg.C_70144) were aligned using MultAlin. Signal peptides (yellow highlight) were predicted using SignalP. B. Individual FasI domain alignment and phylogenetic relationships. All the different FasI domains from Human Periostin (HsPN1-4; Q15063) and BIGH3 (Hs_BGH31-4; Q15582) were aligned with those of the sea anemone homologs using MultAlin. The CRS (Carboxylase Recognition Site, as described in [49]) of the HsPN-1 and HsBIGH3-1 first FasI domains is highlighted. Using the segment of the sequence alignment conserved in all sequences, the best-fitted substitution model was evaluated using ProtTest. Using parameter [LG+G; gamma = 1.755], a Maximum Likelihood tree was determined using phyML. Bayesian phylogenetic tree was calculated using MrBayes 3-1.2. (TIF) [file pgen.1002187.s007.tif]
